# Cosegregation of recombinant chromatids maintains genome-wide heterozygosity in an asexual nematode

**DOI:** 10.1126/sciadv.adi2804

**Published:** 2023-08-25

**Authors:** Caroline Blanc, Nathanaelle Saclier, Ehouarn Le Faou, Lucas Marie-Orleach, Eva Wenger, Celian Diblasi, Sylvain Glemin, Nicolas Galtier, Marie Delattre

**Affiliations:** ^1^ Laboratory of Biology and Modeling of the Cell, Ecole Normale Supérieure de Lyon, CNRS UMR 5239, Inserm U1293, University Claude Bernard Lyon 1, Lyon, France.; ^2^ Institut des Sciences de l'Evolution, Université Montpellier, Institut de Recherche pour le Développement, 34090 Montpellier, France.; ^3^ University of Rennes, CNRS, ECOBIO (Ecologie, Biodiversité, Evolution)–UMR 6553, F-35000 Rennes, France.; ^4^ Department of Ecology and Genetics, Evolutionary Biology Centre, Uppsala University, 75236 Uppsala, Sweden.

## Abstract

In asexual animals, female meiosis is modified to produce diploid oocytes. If meiosis still involves recombination, this is expected to lead to a rapid loss of heterozygosity, with adverse effects on fitness. Many asexuals, however, have a heterozygous genome, the underlying mechanisms being most often unknown. Cytological and population genomic analyses in the nematode *Mesorhabditis belari* revealed another case of recombining asexual being highly heterozygous genome-wide. We demonstrated that heterozygosity is maintained despite recombination because the recombinant chromatids of each chromosome pair cosegregate during the unique meiotic division. A theoretical model confirmed that this segregation bias is necessary to account for the observed pattern and likely to evolve under a wide range of conditions. Our study uncovers an unexpected type of non-Mendelian genetic inheritance involving cosegregation of recombinant chromatids.

## Teaser

Heterozygosity in the asexual nematode *M. belari* is maintained by co-segregation of recombinant chromatids in female meiosis.

## INTRODUCTION

Asexual animal species are composed of females, which produce diploid daughters without paternal genome contribution. Asexuality requires the production of diploid oocytes and, hence, a modified female meiosis. Asexuality, which is derived from sexuality, has emerged multiple times and independently over the course of evolution, and many routes to producing diploid oocytes have been documented (*1*–*3*).

**Fig. 1.**
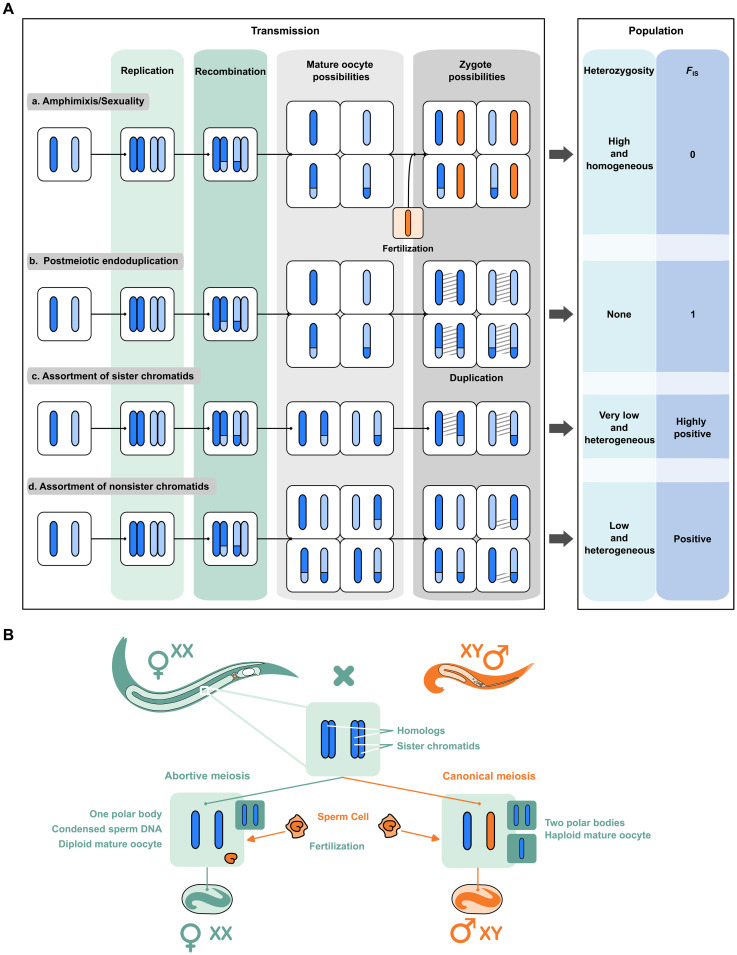
Genetic expectation upon modification of meiosis in asexuals and reproductive system of *Mesorhabditis belari*. (**A**) Description of the expected genetic composition of a zygote and of a population upon different types of modifications of the meiotic program (b to d), often referred to as automixis. By contrast, in amphimictic reproduction, i.e., in regular sexual species, a canonical meiotic division generates a haploid set of maternal chromosomes (in blue), which fuse with the haploid paternal chromosomes (in orange) (a). Each bar represents a chromatid. Dark and light blue correspond to homologous chromosomes in oocytes. (b to d) Upon recombination, assortment of chromatids after modification of meiosis generates stretches of homozygosity, shown with hatching. Consequently, the level of heterozygosity in the population (right column) is decreased. The coefficient of inbreeding (*F*_IS_) measures the excess of homozygosity compared to the Hardy-Weinberg expectation (i.e., in a randomly mating population); positive *F*_IS_ means an excess of homozygotes. Note that assortment of nonsister chromatids (d) is often referred to as “central fusion” (i.e., fusion of the two first products of meiosis), because it is genetically identical, although a fusion of meiotic products is not necessarily the cause of this type of unreduced meiosis. Similarly, assortment of sister chromatids (c) is generally referred to as “terminal fusion.” (**B**) Schematic representation of the reproductive system found in *M. belari* as described in (*13*). Females (in green) produce two types of oocytes. Through canonical meiosis, a single chromatid per chromosome is transmitted to the oocyte (in blue). The sperm provides in single chromatid (in orange). The resulting diploid individuals give rise to males. Ninety percent of the oocytes are however diploid (incomplete meiosis, on the left) in which case the sperm does not contribute DNA because the sperm DNA is set aside after fertilization. These individuals give rise to females.

Depending on the type of cellular modification, different genetic outcomes are expected (Fig. 1A). A common prediction is that most modifications should lead to loss of heterozygosity (LOH). For some species, the entire meiotic program is achieved as in sexual species; however, the final haploid nucleus undergoes a duplication event (gamete duplication), which immediately generates a homozygous individual (Fig. 1A, b). When there is homologous recombination and one of the two meiotic divisions fails, either because the division is abortive or because the products of meiosis fuse back, LOH is also expected either distally or proximally to the crossover location (Fig. 1A, c and d). Hence, maintenance of heterozygosity is theoretically expected only in species for which meiotic recombination is largely reduced or totally abolished (*4*).

LOH is expected to negatively affect fitness because of the exposure of recessive deleterious mutations at diploid state. This has been suggested as a potential cause of the relative scarcity of asexual organisms in nature (*5*) and a selective pressure for reduced recombination rates in asexuals (*6*). Species that maintain some level of heterozygosity do not expose deleterious mutations and as such may circumvent some of the drawbacks of asexuality. In agreement with this expectation, a number of asexual lineages display appreciable amounts of heterozygosity (*7*–*10*). Yet, except in species for which a total loss of recombination has been demonstrated [for instance, (*11*, *12*)], the mechanisms of heterozygosity maintenance are still debated (*7*). Therefore, there is still a need to confront the cytological description and empirical genome data to reach a clear understanding of the genomic and cellular constraints in asexual animals.

We explored the mechanism of meiosis in the auto-pseudogamous nematode *Mesorhabditis belari* (Fig. 1B) (*13*). In this species, a female produces mainly diploid oocytes, which, although fertilized by a sperm, develop only from the maternal DNA and become females. This is also referred to as pseudogamy or sperm-dependent parthenogenesis. The same female also produces ~10% of haploid oocytes through regular meiosis, which, once fertilized, undergo fusion of the parental genomes. These amphimictic diploid embryos will give rise to males because active sperm cells always carry a Y chromosome (*13*). Hence, this species produces 90% asexual females and 10% sexual males. The males are needed to activate the oocytes that will mainly develop as asexual females, and for this reason, this reproductive strategy has been called auto-pseudogamy (*13*).

In this peculiar system, *M. belari* females have most likely maintained recombination for the production of regular oocytes (for the rare males). Given this constraint, we asked which modification of meiosis has been selected to produce the unreduced oocytes (for the asexual females) and with which genomic consequence for the species.

## RESULTS

### Diploid oocytes of *M. belari* are formed after failure of the first meiotic division

**Fig. 2.**
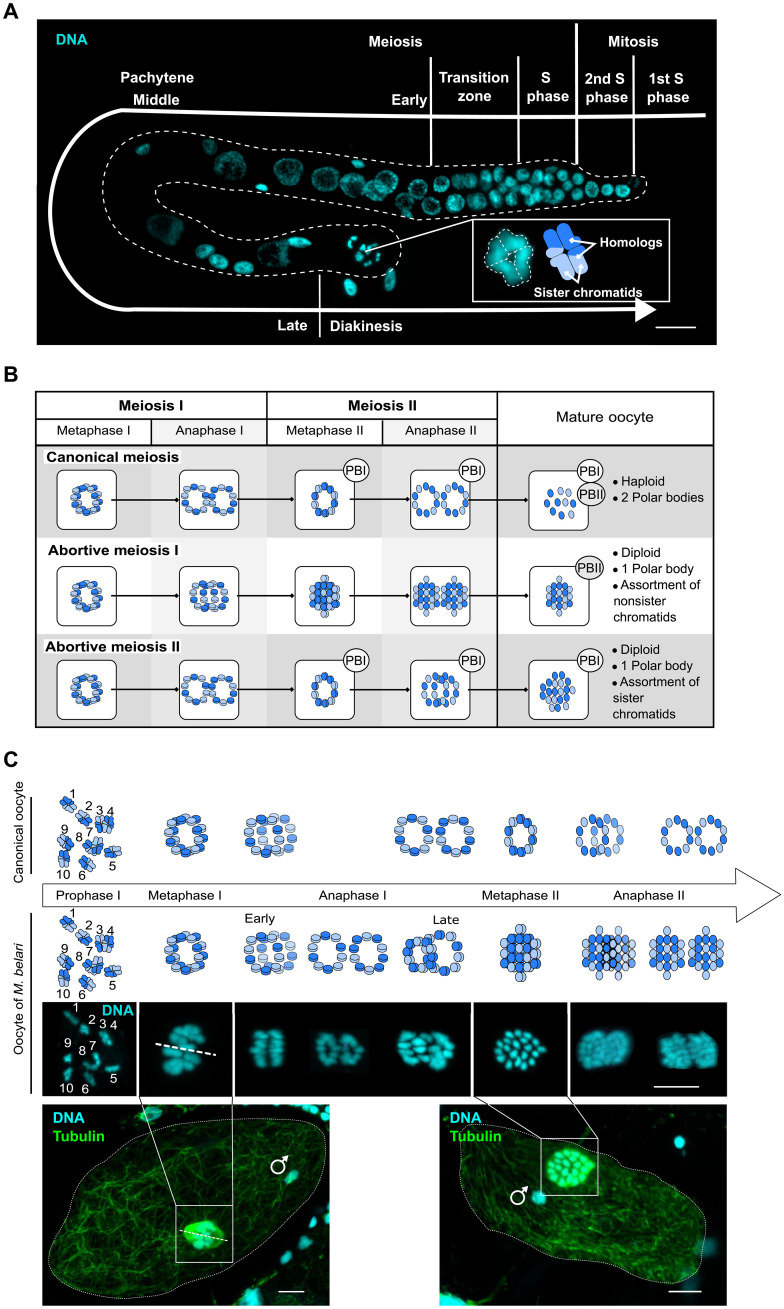
Cytological evidence of abortive meiosis I in *M. belari* females. (**A**) Gonad of an *M. belari* female stained for DNA, showing the progression of meiotic cells along the tract. *M. belari* is diploid, carrying 2*n* = 20 chromosomes. Oocytes in diakinesis are found on the proximal part, with chiasmatic chromosomes. DNA is in blue. Scale bar, 20 μm. The holocentric bivalent chromosomes show a typical cross shape. The bivalents are schematized in blue, one chromosome is in dark blue, and its homolog is shown in light blue. (**B**) Expected patterns of chromosome organization upon failure of meiosis I or meiosis II in *M. belari.* A canonical meiosis is shown on the top. At metaphase, chromosomes orient as a ring. During anaphase, chromosomes (anaphase I) or chromatids (anaphase II) segregate as two rings. PB represents the polar body. (**C**) Reconstitution of *M. belari* meiosis in amphimictic (canonical meiosis) and pseudogamous (incomplete meiosis) embryos from fixed samples. On the bottom, representative pseudogamous embryos from which the images are taken. A sperm DNA is visible, although it will remain condensed and will not fuse with the female DNA. The polar body has not been extruded yet. Tubulin is in green, and DNA is in blue. The dotted line represents the long axis of the meiotic spindle along which the bivalent chromosomes align. Scale bars, 5 μm.

We first determined which steps of meiosis were modified to produce diploid oocytes in *M. belari*. We followed meiosis, making use of the spatiotemporal organization of the gonad, as found in the well-studied *Caenorhabditis elegans* species and other Rhabditidae nematodes (Fig. 2A). We had previously shown that this species is diploid and carries 2*n* = 20 holocentric chromosomes (*13*). First, analysis of oocytes in diakinesis revealed that the 20 chromosomes were always paired into 10 units (*n* > 200 oocytes). Moreover, many bivalents had a crossed-shape structure (Fig. 2A), which resemble the chiasmata of holocentric chromosomes found in *C. elegans* (*14*, *15*), strongly suggesting that chromosomes undergo homologous recombination in *M. belari*.

Next, we reconstructed the different steps of meiotic divisions (Fig. 2, B and C). We found, as is the case for *C. elegans*, that oocytes are arrested at prometaphase of meiosis I, and meiosis resumes after fertilization. The long axis of the bivalents is oriented parallel to the spindle axis, as expected for holocentric chromosomes in regular meiosis (Fig. 2C) (*14*). In the Rhabditidae sexual species studied so far, a polar body is extruded at each meiotic division. The polar bodies are easily recognizable as tiny cells at the edge of the embryo (*16*). We had previously shown that all pseudogamous embryos in *M. belari* (i.e., developing from the unreduced oocytes) had only one polar body demonstrating that one meiotic division was suppressed (*13*). We reasoned that if meiosis I was abortive, the 10 bivalents should dissociate into 20 univalents and no polar body should be detected at this stage. These univalents should next enter anaphase of meiosis II, showing two times 20 DNA-stained bodies (Fig. 2B). In contrast, if meiosis I was successful, a polar body would be extruded, and the 10 univalents would disassemble into 20 units corresponding to 20 sister chromatids after failure of meiosis II (Fig. 2B). We found many cells showing a metaphase plates containing 20 DNA-stained bodies. We also detected anaphase figures with two times 20 DNA-stained bodies (Fig. 2C). None of these cells had produced a polar body. Notably, we also found images of metaphase with only 10 DNA-stained bodies, which we interpret as being either figures of regular meiosis (~10% are expected) or the initial step of meiosis I before abortion. From these results, we concluded that diploid oocytes in *M. belari* are formed after failure of the first anaphase of meiosis I. This modification will lead to the assortment of nonsister chromatids in the oocytes (equational division only). Such type of meiosis is often referred to as “central fusion automixis,” because genetically, it corresponds to the fusion of the two first products of meiosis (Fig. 1A, d). In the presence of recombination for all chromosomes, this pattern of inheritance should progressively lead to LOH, distally to the crossover.

### *M. belari* has a widely heterozygous genome

We analyzed the level and the distribution of heterozygosity in the genome of *M. belari* females, from our laboratory strain JU2817 and nine other wild strains, which had been sampled in different locations around the world (*17*). We sequenced mixed stage animals from each strain and mapped the short reads on the assembled genome of *M. belari* JU2817. We computed genome-wide heterozygosity by counting the proportion of heterozygous positions relative to the total number of positions using Analysis of Next Generation Sequencing Data (ANGSD) (*18*). Each strain being isofemale (see Materials and Methods), the genotype of a strain corresponds to the genotype of a single individual.

We found that all 10 strains had approximately the same level of heterozygosity of about 1.3% (SD = 0.2) (i.e., one residue every 75 nucleotides is heterozygous), demonstrating that the strains behaved similarly in the wild and in the laboratory (fig. S1 and table S1). This level of heterozygosity is unexpectedly high for a meiotic asexual experiencing regular recombination; it is 10 times higher than the self-fertilizing nematode *C. elegans* (*19*) and similar to natural populations of the fruit fly *Drosophila melanogaster* (*20*), for instance. Heterozygosity could be maintained in most parts of the chromosomes and lost only in subtelomeric regions if crossovers were restricted to chromosome ends, but we found that heterozygosity was uniform along all contigs (fig. S1). Analyzing this heterozygosity in the wild strains, we revealed the inbreeding coefficient *F*_IS_ being close to 0, i.e., *F*_IS_ = 0.019, throughout the genome. In a randomly mating species (Hardy-Weinberg expectation), *F*_IS_ equals 0. *F*_IS_ is negative if there is an excess of heterozygosity compared to the Hardy-Weinberg expectation, as found in asexuals that have lost recombination, via the so-called Meselson effect (*21*, *22*). *F*_IS_ is positive if there is a deficit of heterozygosity, as in selfers for instance (*23*). We had previously shown that out of 1000 females, no sexual females were produced (*13*). Such a *F*_IS_ value was therefore unexpected for a species with a rate of sexual reproduction close to 0 (Fig. 1A).

To further confirm the maintenance of heterozygosity, we performed a genome-wide analysis of genotype inheritance, from mother to daughters in *M. belari* JU2817. We performed this analysis on the transcriptome, which can be easily obtained from single worms. We analyzed three female individuals descended from the same mother. We mapped the RNA-sequencing (RNA-seq) reads to the previously assembled *M. belari* JU2817 transcriptome, and we called genotypes, focusing on sufficiently covered contigs and positions. Under the assumption of active recombination and random segregation of chromatids, large chromosomal segments—and therefore a substantial number of single-nucleotide polymorphisms (SNPs) and contigs—are expected to be homozygous in some of the females (Table 1). In contrast to this prediction, we found only a small minority of SNPs for which at least one daughter had a homozygous genotype (Table 1). These SNPs were most often surrounded by SNPs for which all three daughters were heterozygous, suggesting that there was no real stretch of homozygosity in any female. Among the >3200 contigs with more than two SNPs, only 9 contigs carried >2 SNPs homozygous in the same daughter. We found similar proportions when we performed the same analysis in another auto-pseudogamous species, *Mesorhabditis monhystera*. As a control, we also analyzed two sexual *Mesorhabditis* species *M. longespiculosa* and *M. spiculigera*. In these species, we found a large fraction of SNPs with homozygous genotypes (Table 1), as expected under random mating of gametes, with 737 (out of 1423, ~52%) and 1371 (out of 2953, ~46%) contigs, respectively, carrying >2 SNPs homozygous in the same daughter. This analysis indicates that the modified meiosis in auto-pseudogamous species of *Mesorhabditis* could fully preserve heterozygosity, from one generation to the next, and in the population overtime.

**Table 1.** Patterns of shared heterozygosity among sisters in four species of *Mesorhabditis*.

	Pseudogamous	Pseudogamous	Sexual	Sexual
Species	*M. belari*	*M. monhystera*	*M. spiculigera*	*M. longespiculosa*
No. of contigs	8381	8632	7099	7564
Mean coverage	37×	28×	42×	40×
No. of polymorphic sites	34,867	29,664	43,543	20,243
One-heterozygote*	1.44%	0.42%	45.3%	23.5%
Two-heterozygote^†^	0.89%	0.99%	22.1%	27.6%
Three-heterozygote^‡^	97.5%	98.5%	22.4%	39.4%
Other^§^	0.1%	0.06%	10.2%	9.5%

*Proportion of polymorphic sites at which exactly one of the three sisters was heterozygous.

†Proportion of polymorphic sites at which exactly two of the three sisters were heterozygous.

‡Proportion of polymorphic sites at which all three sisters were heterozygous.

§No heterozygote or unexpected allele call.

These results seem to contradict our initial cytological observations. We therefore determined whether despite the presence of structures that resemble chiasmata, homologous recombination might be absent, which would then explain the maintenance of heterozygosity in the short and long term upon assortment of nonsister chromatids.

### Homologous chromosomes recombine during female meiosis

**Fig. 3.**
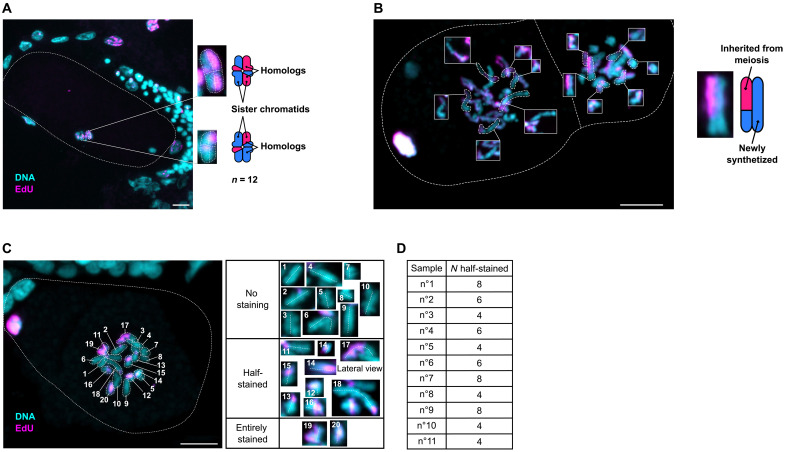
Evidence of recombination and cosegregation of recombinant chromatids. Fixed embryos after an EdU experiment, where one chromatid out of the two is stained with EdU (in pink). DNA is in blue. Scale bar, 5 μm. (**A**) Embryo in metaphase of meiosis I (i.e., no polar body has been extruded yet). The metaphase plate is perpendicular to the glass slide. Few chromosomes are visible on this lateral view. The expected exchange of chromatids, as described in (*24*), is shown as well as the actual images in the inset. (**B**) Two-cell stage embryo during prometaphase. Most chromosomes have a bicolor chromatid (half-blue/half-pink, demonstrating recombination) and an unlabeled chromatid because it has replicated during the previous S phase in the absence of EdU (entirely blue). (**C**) One representative one-cell embryo in prometaphase. All 20 chromosomes are shown in the insets. Eight chromosomes are bicolor (#14 is shown twice). In (B) and (C), the polar body is visible on the left and contains EdU-labeled chromatids, as expected. (**D**) Table summarizing the count of recombinant chromatids from 11 embryos (also shown in fig. S3).

We wished to directly visualize crossovers as a formal proof that homologous recombination occurs during *M. belari* female meiosis. To this end, we used the thymidine analog 5-ethynyl-2'-deoxyuridine (EdU), which is incorporated into replicating DNA and can be fluorescently labeled. *M. belari* females were bathed in EdU (pulse phase) and then allowed to recover (chase phase) so that germ cells entered mitotic cycles with EdU and next divided and replicated without EdU. We optimized the pulse and chase periods to obtain chromosomes harboring only one EdU-labeled chromatid (fig. S2). Upon recombination, we then expected a strain exchange between one EdU-labeled (shown in pink) and one non–EdU-labeled chromatid (shown in blue, labeled only with Hoechst) in 50% of cases, generating bicolor chromatids (blue/pink) (Fig. 3 and fig. S2). We first analyzed the color of chromatids in diakinesis oocytes in which homologous chromosomes form chiasmata, i.e., bivalents. The expected figures of crossover in holocentric chromosomes has been described in (*24*) and is depicted in Fig. 3A. From eight oocytes, we identified 26 bivalent chromosomes whose orientation allowed us to unambiguously distinguish the chromatids within the chiasma. For 12 of them, the two opposed chromatids had the same color and could not be analyzed. Among the 14 showing opposed chromatids of different colors, 13 bivalents showed an exchange of chromatids, and only 1 showed no exchange (Fig. 3A). We also analyzed chromosomes in the female pronuclei of pseudogamous eggs during the first or second cell cycle, when chromosomes are condensed and chromatids are clearly visible. Cycles of DNA replication and mitosis had occurred in the absence of EdU in embryos, generating many chromatids devoid of EdU. Nevertheless, we counted 49 bicolor chromatids from 12 embryos (one representative embryo is shown in Fig. 3B). This analysis also revealed that chromatid exchange is not restricted to chromosome ends, as many chromosomes show large portions of EdU-positive chromatids (Fig. 3B). These results demonstrate that exchanges of strands between homologous chromosomes during meiosis are frequent and that recombination, not restricted to telomeric regions, does occur in *M. belari* during the production of unreduced oocytes.

As another evidence that recombination is maintained in *M. belari*, we analyzed patterns of linkage disequilibrium (LD) across loci. In the absence of sex and recombination, alleles are expected to be strongly associated among loci, with haplotype blocks extending over long stretches of DNA. Recombination, if at work, breaks allele associations, leading to a decay of LD as the physical distance between SNPs increases (*25*). To assess the extent of LD in *M. belari*, we analyzed the genomic data previously obtained from the 10 strains. We called SNPs and used the LDhelmet program to estimate the genome-wide distribution of the effective population recombination rate ρ. This analysis indicated that, in both species, the estimated ρ was homogeneous across the genome with no local enrichment or losses (fig. S1), with a point estimate of the average ρ of 0.038 per base pair (bp). This implies that LD is lost as the distance between the considered loci exceeds ~100 bp. The average estimated ρ in *M. belari* was similar to estimates reported in sexual species of arthropods, such as *D. melanogaster* (*20*), and indicative of a high effective population recombination rate in this auto-pseudogamous species.

### Chromatid segregation is biased during the unique meiotic division of *M. belari* embryos

Our cytological and genomic data are contradictory because recombination and random assortment of nonsister chromatids should lead to LOH. We reasoned that maintenance of heterozygosity from mother to daughters, and at the population level, can be achieved despite recombination, if either the two recombinants or the two nonrecombinant chromatids of a given chromosome pair cosegregate into the egg during the unique division of meiosis. We validated this hypothesis using our EdU experiment. Because *M. belari* chromosomes cannot be distinguished cytologically (i.e., pairs cannot be recognized), we used a statistical approach. We reasoned that under the hypothesis of cosegregating recombinant chromatids, we should always find an even number of recombinant chromatids in the nuclei of one-cell stage embryos, before the first mitosis. We reanalyzed another set of one-cell embryos, selecting only those in which chromosomes were well spread out at prometaphase so that chromosome axis was unambiguously identified. In the 11 embryos analyzed, we always found an even number of bicolor chromatids, ranging from 4 to 8 (Fig. 3C and fig. S3). Such a pattern would be obtained very rarely in the case of random segregation of the 20 chromatids (*P* value = 0.00048, binomial test, *n* = 11, *P* = 0.5). This result strongly supported that the single meiotic division of *M. belari* females is unique, as it leads to the cosegregation of recombinant chromatids (CRC).

### Modeling the reproductive strategy of *M. belari* and the cosegregation of recombinant chromatids during meiosis

To assess whether CRC could reconcile cytological and genomic data, we developed a population genetics model. As output parameters, we considered the level of heterozygosity, the LD (as measured above), and the inbreeding coefficient *F*_IS_.

**Fig. 4.**
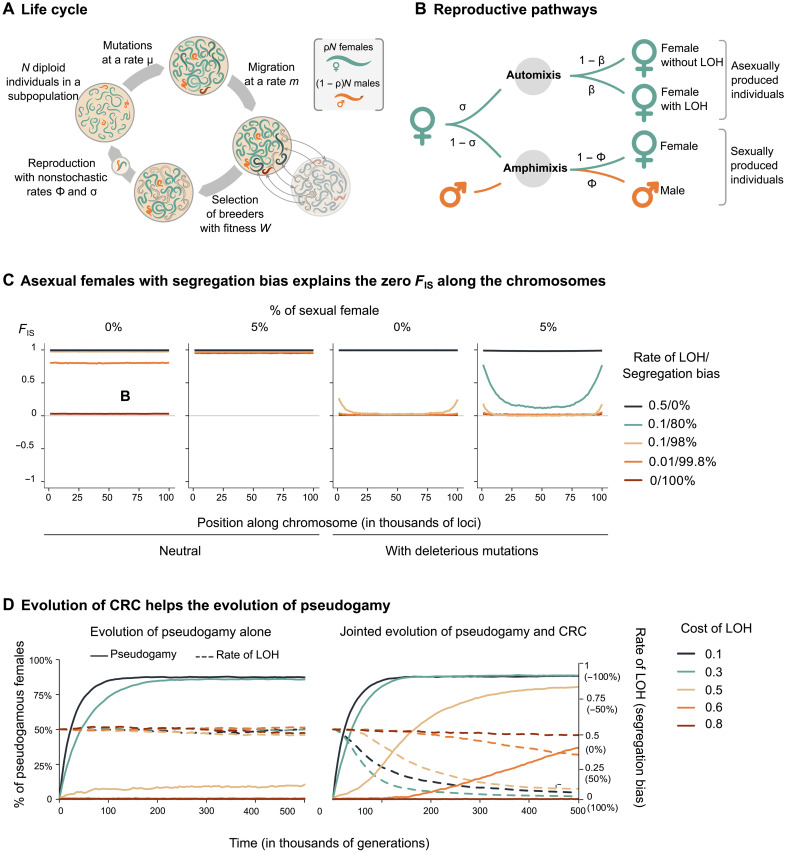
Modeling the genomic consequences and evolution of CRC in *M. belari.* (**A**) Simulated life cycle with associated parameters. The generations are discrete and nonoverlapping, and we assume an island model to simulate the high level of consanguineous mating: The lower the local population size (*N*) and the migration rate (*m*), the higher the level of consanguineous mating. (**B**) Reproduction occurs either by automixis (i.e., producing asexual females) at rate σ or by amphimixis at rate 1 − σ. During automixis, the rate of LOH is β. With *b*, the cosegregation bias of recombinant chromatids, $\beta =\frac{1}{2}(1-b)$. With no bias (*b* = 0) (i.e., random association of chromatids), β = ½, whereas with a full segregation bias (*b* = 1), there is no LOH. Note that complete LOH, β = 1, corresponds to negative cosegregation, when one recombinant always segregates with one nonrecombinant chromatid. Under sexual reproduction, females are produced at rate ϕ. ϕ is close to zero in natural populations. σ and ϕ determines the sex ratio, ρ. (**C**) *F*_IS_ along a chromosome as a function of the segregation bias and corresponding LOH, with or without sexually produced females and deleterious mutations. The total population size is 1000, and the migration rate is *m* = 0.0005. (**D**) Evolution of pseudogamy from an initially sexual population with different costs of LOH when the rate of LOH is fixed (β = ½), or when it is allowed to evolve through CRC. We assume 10% of males as in natural populations. Allowing the sex ratio to evolve does not change the result. The proportion of pseudogamous females (plain lines) reaches 90% when there are no more sexual females. The corresponding rate of LOH/segregation bias evolving with CRC is shown with dashed lines. Note that mutants increasing LOH (through a negative bias) are introduced in the simulations but are never selected for.

We modeled the life cycle of *M. belari*, including the production of sexual males and asexual females with a biased sex ratio, the inbreeding mating structure [brother-sister mating or strong family structure as proposed by Grosmaire *et al.* (*13*)], and the modified meiosis with variable rate of LOH due to variable bias in the segregation of chromatids (Fig. 4, A and B). We also allowed for rare production of sexual females (i.e., being produced after mixing of the parental genomes). Although no sexual females have been observed under laboratory conditions, they may exist at low rate in natural populations, and it is an intermediate stage that necessarily occurred in the transition from sexuality to asexuality. We looked for conditions that could explain the observed genomic pattern: high heterozygosity, *F*_IS_ ~ 0, and low LD.

First, analytical results and multilocus simulations confirmed that CRC is needed to explain the absence of LOH. Second, considering deleterious mutations throughout the genome broadens the conditions that can explain the observed genomic patterns: Strict CRC is not required and sexual females can be produced at low rate (Fig. 4C and Supplementary Text). Actually, the production of sexual females at a very low rate better explains the low and flat LD pattern, as well as the *F*_IS_ ~ 0 than pure asexuality (Supplementary Text). The comparison of results without and with deleterious mutations also illustrates the central role that recessive deleterious mutations likely play in the system. Highly homozygous individuals that should be produced by imperfect CRC or leaky sex (that should lead to *F*_IS_ > 0; Fig. 4C neutral) are selected against, maintaining *F*_IS_ close to zero for a large range of conditions (Fig. 4C, with deleterious mutations). This also supports the idea that LOH should be costly and suggests that CRC could be selected as a LOH-preventing mechanism.

We tested this hypothesis via a modification of the initial model whereby CRC can evolve. We first simulated a sexual species with random chromatid segregation at meiosis and added a locus controlling the proportion of asexual females produced (Fig. 4D, left). We assumed one mandatory crossover per chromosome. Hence, the asexual females experienced LOH with associated fitness reduction due to the expression of recessive deleterious mutations in homozygotes, a form of inbreeding depression. If inbreeding depression was higher than 0.5, it compensated for the advantage of not producing males and pseudogamy could not evolve. If inbreeding depression was lower than 0.5, pseudogamy rapidly evolved. Next, we introduced mutations at a second locus controlling CRC during asexual meiosis (in both directions: recombinants could be more positively or more negatively associated than at random) (Fig. 4D, right). We found that mutations leading to positive association between recombinants were selected for and that the population rapidly evolved toward complete CRC, preventing the deleterious effects of LOH. We also found that when mutations affecting pseudogamy and CRC were introduced at the same time, the two mechanisms co-evolved, speeding up and broadening the conditions for the evolution of pseudogamy (Fig. 4D and Supplementary Text). On the one hand, the occurrence of some asexual females enabled the evolution of CRC. On the other hand, once CRC started to evolve, it partly prevented the deleterious effect of LOH, further favoring the evolution of pseudogamy, even when inbreeding depression was higher than 0.5 (Fig. 4D). Our modeling approach thus confirmed that all a priori contradictory observations can be reconciled with the mechanism of CRC during the unique meiotic division of females, and our model provides a selective explanation for the evolution of such a peculiar mechanism from a sexual ancestor.

## DISCUSSION

**Fig. 5.**
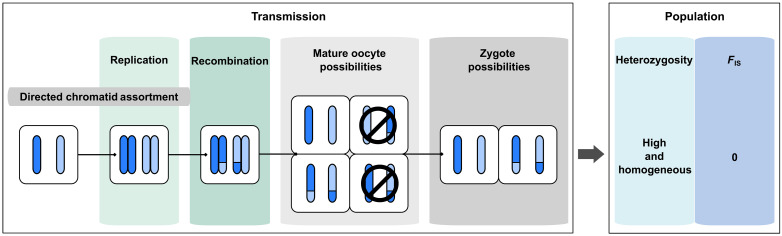
Mechanism of heterozygosity maintenance via CRC in *M. belari.* Schematic representation of meiosis and CRC during the production of diploid oocytes in *M. belari*. Homologous chromosomes (in blue) are initially heterozygous in the mother (shown in nuances of blue). After recombination and failure of meiosis I, nonsister chromatids do not segregate randomly during meiosis II. Instead, cosegregation of recombinant chromatids maintains heterozygosity in the progeny and in the population.

In this study, we found that *M. belari* asexual females are produced in the presence of recombination and assortment of nonsister chromatids, which should lead to rapid LOH, distally to the crossover. Our genomic analysis, however, revealed an unexpectedly high level of heterozygosity throughout the genome and no sign of LOH, even locally. Using a combination of cytological, genomic, and modeling approaches, we demonstrated that this pattern is possible provided the recombinant chromatids of each chromosome pair are not randomly assorted but instead cosegregate during the unique meiotic division. We named this type of non-Mendelian inheritance CRC. With CRC, specific pairs of chromatids are chosen during cell division such that the whole set of maternal alleles is transmitted to offspring (Fig. 5).

During their reproductive lifetime, *M. belari* females produce 10% reduced oocytes via regular meiotic divisions, which develop into sexual males, males being essential for sperm-dependent parthenogenesis (*13*). Hence, the meiotic program is intact in this species. This is a constraint for the evolution of asexuality because upon recombination, asexual females should experience LOH. We discovered CRC as an unexpected mechanism of LOH avoidance despite recombination.

How did CRC emerge during the evolution of auto-pseudogamy in *Mesorhabditis*? One hypothesis is that, initially, some females were produced sexually (i.e., after mixing of the parental genomes) when an X-bearing sperm fertilized the oocytes. Some females were also produced asexually through pseudogamy, after abortion of meiosis I but without CRC. In this context, asexual females experienced LOH, but this was compensated by the heterozygosity of sexual females. Once CRC appeared, LOH was prevented in the asexuals, next allowing the loss of most sexual females. Alternatively, a single modification of the meiotic program could have simultaneously generated a defect in anaphase I and a biased segregation of chromatids, immediately establishing asexual females without LOH.

At this stage, it is difficult to speculate on the molecular mechanism involved, and on a sequence of events, because, to our knowledge, no such phenotype has been described in mutants of model species. Nevertheless, we hypothesize that failure of joint molecule resolution during crossover could maintain the two recombinant chromatids physically attached. Consequently, this attachment would prevent the correct segregation of bivalents during meiosis I. Several nucleases involved in crossover resolution, such as MUS-81 and SLX-4 of XPF-1 (*26*, *27*) or LEM-3/Ankle1, which eliminates the persistent DNA linkages during meiotic II in *C. elegans* (*28*), could be involved. We hypothesize that some of these factors could be down-regulated or have a delayed activity in the oocytes experiencing an incomplete meiotic division in *M. belari*. In contradiction with this hypothesis, we have not been able to detect DNA threads between the separating chromosomes. However, thin DNA threads are most often not detectable with conventional DNA staining as we have used in this study but only with immunofluorescent staining for proteins that bind these threads (*29*). A systematic analysis of the dynamic localization of the nucleases involved in crossover resolution in *M. belari* may help determine whether or not a persisting physical link is responsible for CRC. An alternative hypothesis is that despite proper crossover resolution and loss of a physical attachment, some of the proteins that are differentially loaded on chromatids during meiotic prophase remain specifically attached to the recombinant chromatids. Such “tagging” could contribute to a directed rotation of chromatids during meiosis II, with recombinant facing the same pole of the spindle. Exploration of *M. belari* mutants affecting the different steps of crossover establishment and resolution will be necessary to shed light on the mechanisms at the origin of CRC.

Another mechanism of LOH avoidance has been previously proposed for recombining asexuals, which relies on distal crossover location (*6*, *30*). However, distal crossovers are very unstable for the *C. elegans* holocentric chromosomes and often lead to aneuploidy (*31*), suggesting that such mechanism of LOH avoidance could not have been selected in a holocentric species such as *M. belari*. Another mechanism involving inverted meiosis (which is compatible with holocentricity), failed meiosis II, and biased chromatid segregation has been proposed for the maintenance of heterozygosity in the asexual oribatid mites (*32*, *33*). Although such biased segregation of chromatids remains hypothetical in the absence of further cytological description, it is conceptually similar to the CRC we describe in our study.

If distal COs are most likely not compatible with holocentric species, can CRC evolve as a mechanism of LOH avoidance in asexual species with monocentric chromosomes? We suspect that CRC is not necessarily restricted to holocentric species. In holocentric chromosomes, the CO serves as a focal point to establish the chromosome subdomains, necessary for the sequential dissociation of cohesins and, hence, chromatids (*34*). Therefore, even in the absence of a single centromere, the same principles of chromosome organization and dissociation hold in monocentric and holocentric species. Consequently, co-orientation of recombinant chromatids could also be at play in monocentric species. However, in species that establish multiple crossovers between all chromatids within a pair of homologs (*35*), which is restricted to monocentric species, there will be no possibility to cosegregate the recombinants together. Hence, CRC can probably emerge only in species that initially have a limited number of crossovers per chromosome, as previously proposed (*6*), or at least multiple crossovers restricted to a single pair of chromatids.

Although different from CRC, evidence of biased segregation of recombinant chromatids already exists in monocentric species. For instance, in *Drosophila*, during mitotic recombination, the recombinant chromatids of a given pair of chromosomes systematically segregate away in the daughter cells, which is the opposite to the CRC bias (*36*). In human oocytes, after the two regular steps of meiosis, the recombinant chromatids are preferentially inherited in the oocytes (*37*). Here as well, the mechanisms behind the segregation bias remain to be determined, but together with CRC, these examples show that recombinant and nonrecombinant chromatids can be sorted out during cell divisions.

Last, most asexual animal models have been characterized either by cytology or by genomics, rarely both, whereas such a combination of approaches was decisive here. In most cases, the level of recombination in the asexual lineages was inferred from the observed rate of LOH. For the Cape honey bee, the crustaceans *Artemia parthenogenetica* and *Daphnia magna* or the ant *Cerapachys biroi* to only cite a few, a reduction in the rate of recombination has been invoked to account for the observed level of elevated heterozygosity and absence of LOH (*6*, *30*, *38*, *39*). In the light of our discovery in *M. belari*, we would like to propose CRC as a possible alternative explanation. Overall, there is an exciting possibility that CRC has been selected multiple times independently in the evolution of asexuals, as an efficient strategy against the detrimental effect of LOH.

## MATERIALS AND MEHODS

### Nematode strains and culture

*Mesorhabditis* species are maintained at 20°C on Nematode Growth Media (NGM) plates seeded with *Escherichia coli* OP50, following *C. elegans* protocols, as described in (*13*).

### Immunostainings on gonads and embryos

We performed immunostainings as described in (*13*). We dissected gravid females on slides coated with 0.25% poly-lysine in 0.5× M9. After freeze cracking, we fixed the samples by immersing slides into methanol at −20°C for at least 5 min. We used a mouse anti-tubulin antibody as primary antibody (1:2000; Sigma-Aldrich, DM1A) and an Alexa Fluor 488 donkey anti-mouse secondary antibody (1:2000; Jackson ImmunoResearch, #715-545-150). We incubated both antibodies at room temperature for 45 min. DNA was stained using Hoechst 33258 at 0.5 μl/ml (Merck Sigma-Aldrich, #94403). Images were acquired using a confocal microscope (oil immersion 63× objective, LSM800 and LSM980 Airyscan, Zeiss). *Z*-stacks of embryos were acquired every 0.15 μm. Last, we treated the acquired images using the ImageJ 1.53t software.

### EdU pulse/chase protocol

We adapted the protocol from (*15*). To obtain diakinesis oocytes and early embryos for which only one chromatid per chromosome was labeled with EdU, we had to optimize the protocol for *M. belari*. First, we synchronized worms using axenization, as described in (*13*). Briefly, we collected worms and treated them with bleach and NaOH to dissolve all individuals except the embryos. We washed the embryo pellet and placed it on plates without bacteria, allowing L1 larvae to hatch. Without food, all L1s were arrested at the same stage after 2 days. We placed L1s back on food and allowed them to grow for 72 hours at 20°C. At this stage, we collected the young L4 synchronized worms and washed them in 1× phosphate-buffered saline (PBS) and 0.1% Triton X-100. We transferred a pellet of ~300 μl of worms into 200 μl of 10 mM EdU diluted in water (Thermo Fisher Scientific, A10044) to obtain a final concentration of 4 mM EdU. We transferred the tube on a rotator for 4 hours at room temperature. After washes in M9, we plated the animals onto fresh NGM plates seeded with *E. coli* and placed them at 25°C for 48 hours before embryos were collected for fixation. As summarized in fig. S2, we deduced that two rounds of S phase precede meiotic prophase during *M. belari* oogenesis.

### EdU click-it labeling

Cytology was performed following the instructions provided by the EdU Click-it kit (Thermo Fisher Scientific, C10337). We collected the embryos after axenization, as described above. We placed the embryos on poly-lysine–coated slides and freeze-cracked and fixed them in −20°C methanol. We incubated the samples with bovine serum albumin 2% for 20 min at room temperature. We washed the slides twice with 1× PBS. Following the instructions of the kit, we washed the slides for 30 min at room temperature in 1× PBS with 1% Triton X-100 (v/v) and labeled them with Alexa Fluor 488–azide for 30 min at room temperature. We washed the samples twice with 1× PBS and incubated them in a tank with Hoechst 33258 for 20 min at room temperature. Last, we mounted the slides using ProLong Diamond Antifade Mountant (Thermo Fisher Scientific, P36965) and sealed them with nail polish. For scoring the segregation of recombinant chromatids, we first screened the slides for one-cell stage embryos in prometaphase, because at this stage, the chromosomes are well condensed and the chromatids can be easily visualized. Among these embryos, we first selected the embryos for which the EdU signal is present on the DNA but is not uniform (which happens if the EdU was present during too many S phases). Second, we scored only those embryos for which the 20 chromosomes were nicely spread, to be confident about the color of the chromatids. For this reason, the number of analyzed embryos remains limited. This is a technical limitation of the study system because with these nematode embryos, we cannot synchronize the embryos to increase the number of cells in prometaphase and we cannot perform metaphase spread because of the eggshell surrounding the cells.

### Airyscan and ImageJ 3D analysis of EdU-labeled recombinant chromatids

As with immunostaining, confocal airyscan images were acquired using the Zeiss LSM800 Airyscan and LSM980 Airyscan using a 63× oil objective and a 0.15-μm interval between slides. Images were subsequently processed by the airyscan processing method [three-dimensional (3D) analysis, automatic low stringency, Zen Blue 3.3]. Processed images were treated using ImageJ 1.53t software. Analyses of each chromosome were done using a combination of Z projection and 3D projection on the *y* axis using the brightest point method and interpolation.

### DNA and RNA preparation for sequencing

We performed DNA sequencing on 10 strains of the auto-pseudogamous species *M. belari* coming from different locations in Europe (accession numbers PRJEB30104 and PRJEB61636). For each strain, one gravid female was initially collected in the wild and left to lay eggs in a Petri dish. This constituted a single strain, which was frozen in our collection. For sequencing, we amplified the animals and extracted the DNA for each strain. Briefly, we collected mixed stage worms, washed them in M9, and we froze a pellet of ~300 μl of worms in liquid nitrogen. After thawing, we added 600 μl of cell lysis buffer (Qiagen Cell Lysis Solution, #158906), as well as 6 μl of proteinase K at 17 μg/μl and incubated the mix for 3 hours at 65°C. We next incubated the mix for 1 hour at 37°C, supplemented with 40 μl of ribonuclease A (at 5 mg/ml). We added 200 μl of protein precipitation solution (Qiagen, #158912), and after 5 min on ice, we centrifuged the mix for 10 min at 13,000 rpm at 4°C. We next added 600 μl of isopropanol to the supernatant. After 10 min at room temperature, we centrifuged the mix at maximum speed, and we rinsed the pellet twice in ethanol 70°C. We then dried the pellet, and resuspended it in nuclease-free water.

For the analysis of genotype inheritance in sisters, we isolated gravid females, for each species, let them lay eggs, and, after few days, isolated three virgin daughters. We extracted the mRNAs of each single female using the SmartSeq2 protocol, as described in (*40*). For all samples, we prepared genomic libraries (insert sizes of ~550 bp) using TruSeqNano, and we sequenced the libraries on a HiSeq4000 with 100-bp paired-end read length.

### Heterozygosity analysis

For each of the 10 strains of *M. belari*, we mapped the reads to the assembled genome of *M. belari* (accession number PRJEB30104) with BWA (Burrow-Wheeler Aligner) (*41*). The assembled genome is still fragmented, containing 217 contigs. We therefore analyzed heterozygosity along all 217 contigs (see fig. S1 and zenodo archive; https://doi.org/10.5281/zenodo.8112093). We produced BAM files with SAMtools (*42*), and we estimated the heterozygosity for each strain using ANGSD (*18*) using the Site Frequency Spectrum (SFS) estimation for a single sample. Recombination at the extremities of the chromosomes could explain a limited decrease in heterozygosity, with the rest of the chromosome remaining nonrecombining. To test this, we calculated the heterozygosity on 5000-bp windows. A decrease in heterozygosity at the ends of the contigs was then looked for, graphically. At first, we detected homozygous portions in the JU2817 strain. However, these portions were twice as low in coverage as the rest of the contigs, which could be explained by an assembly error due to too much divergence between the two alleles. A similarity search with blastn allowed us to detect the presence of indels between the two alleles, preventing a unique assembly of these regions. We did not find this pattern in the other strains and therefore explains the lower heterozygosity of this strain compared to the others (i.e., haplotype divergence).

### Genotype inheritance

RNA-seq was performed for three sisters in each of the two auto-pseudogamous species *M. belari* and *M. monhystera* and the two sexual species *M. spiculigera* and *M. longespiculosa* (accession number PRJEB61636). For each species, reads from the three individuals where pooled to assemble a transcriptome: Adapters were clipped from the sequences, low-quality read ends were trimmed (phred score < 30), and low-quality reads were discarded (remaining length <36 bp) using trimmomatic [v0.39; (*43*)]. Paired-end transcriptomes were de novo assembled using Trinity v2.13.2 (*44*). We mapped the reads on their respective assembled transcriptome with BWA (*41*), and we produced the BAM files with SAMtools (*42*) and called the SNPs using reads2SNP (*45*) focusing on sufficiently covered contigs (minimum contig average coverage = 15×) and positions (minimum = 20×). In each species, we selected positions in which not only the called genotypes but also read frequencies varied significantly among sisters. Specifically, for each position, two multinomial models were fitted to read counts. Model M0 (three degrees of freedom) assumed a common frequency of A, C, G, and T in the three sisters. Model M1 (9 degrees of freedom) rather allowed each of the three sisters to have its own frequencies of A, C, G, and T reads. We performed a likelihood ratio test, and we analyzed only positions in which M0 was rejected (*P* < 1.0 × 10^−8^). This was intended to exclude positions for which the genotype varied among sisters due to uncertainty in genotype calling.

### Measure of LD

To test for the existence of recombination, we estimated the LD using the 10 strains of *M. belari*. For each of the 10 strains, we mapped the reads to the assembled genome with BWA (*41*). We produced BAM files with SAMtools (*42*), and we called the SNPs with reads2SNP (*45*). To phase haplotypes, we first used WhatsHap (*46*) to extract the phase information contained in reads. We completed phasing using Beagle v5.3 (*47*). We computed LD on 5000-bp windows with LDhelmet v1.10 (*20*) using recommended parameters.

## Supplementary Materials

### This PDF file includes:

Figs. S1 to S9 Table S1 Supplementary text Legends for data S1 to S3 References
