# Supplementary material for: Cosegregation of recombinant chromatids maintains genome-wide heterozygosity in an asexual nematode
Source: Sci Adv. 2023 Aug 25;9(34):eadi2804. doi: 10.1126/sciadv.adi2804 (PMC10456839; doi:10.1126/sciadv.adi2804)
Supplement: Supplementary file 1 — Figs. S1 to S9 Table S1 Supplementary text Legends for data S1 to S3 References [file sciadv.adi2804_sm.pdf]

Science Advances
AAAS

Supplementary Materials for

Cosegregation of recombinant chromatids maintains genome-wide heterozygosity in an asexual nematode

Caroline Blanc *et al.*

Corresponding author: Marie Delattre, marie.delattre@ens-lyon.fr

Sci. Adv. **9**, eadi2804 (2023)
DOI: 10.1126/sciadv.adi2804

The PDF file includes:

Figs. S1 to S9
Table S1
Supplementary text
Legends for data S1 to S3
References

Other Supplementary Material for this manuscript includes the following:

Data S1 to S3

Fig. S1

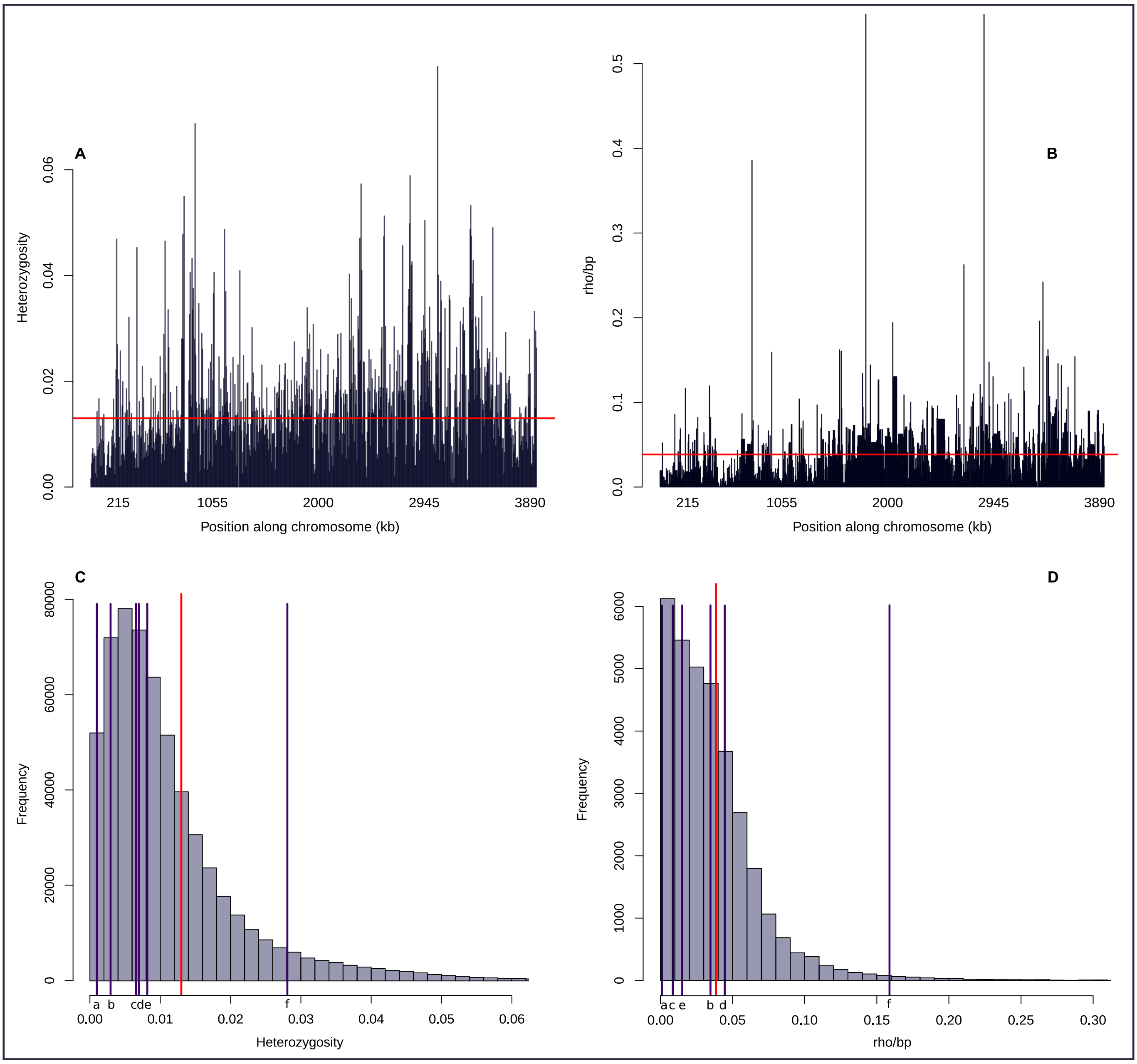
A
B
C
D
Heterozygosity
0.00
0.02
0.04
0.06
215
1055
2000
2945
3890
Position along chromosome (kb)
rho/bp
0.0
0.1
0.2
0.3
0.4
0.5
Position along chromosome (kb)
Frequency
0
20000
40000
60000
80000
a b cde f
0.00 0.01 0.02 0.03 0.04 0.05 0.06
Heterozygosity
Frequency
0
1000
2000
3000
4000
5000
6000
ac e b d f
0.00 0.05 0.10 0.15 0.20 0.25 0.30
rho/bp

Figure S1: **Quantification of heterozygosity and estimation of ρ**.
Heterozygosity (A) and recombination (B) estimated along a representative contig (contig 110) and distribution of heterozygosity (C) and recombination rates (D) computed on 5000bp windows. Genome-wide average heterozygosity and recombination rate ρ are represented by a red bar. Other sexual species, taken from the literature, are depicted with a blue bar: a) *Homo sapiens* (*38*, *39*); b) *Gasterosteus aculeatus* (*40*); c) *Mus musculus* (*41*, *42*); d) *Drosophila melanogaster* (*20*); e) *Taenopygia guttata* (*43*); f) *Heliconius melpomene* (*44*, *45*).
The assembly comprises 218 contigs, a single representative is shown here. The rho and heterozygosity values for windows of 5000 base pairs for each of the 218 contigs are available in the zenodo archive: https://doi.org/10.5281/zenodo.8112093

Fig. S2

A

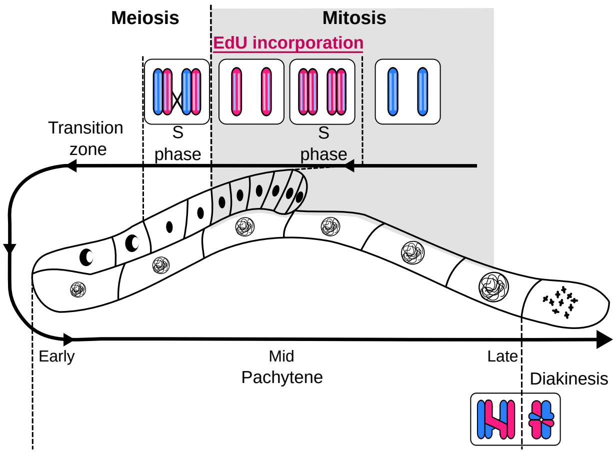

Meiosis
Mitosis
EdU incorporation
Transition zone
S phase
S phase
Early
Mid
Pachytene
Late
Diakinesis

B

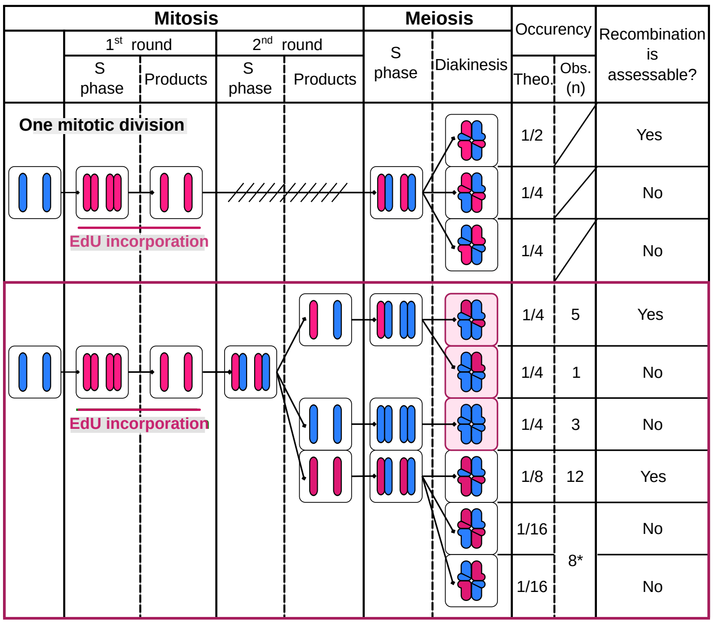

Mitosis
Meiosis
Occurency
Recombination is assessable?
1st round
2nd round
S phase
Products
S phase
Products
S phase
Diakinesis
Theo.
Obs. (n)
One mitotic division
EdU incorporation
1/2
Yes
1/4
No
1/4
No
1/4
5
Yes
1/4
1
No
1/4
3
No
1/8
12
Yes
1/16
No
8*
1/16
No
EdU incorporation

Figure S2: **Design and expectations for the Edu experiment**.
A) Design of EdU pulse-chase method. EdU is pink and DNA is blue. Sketch of *M. belari* gonad showing a pair of homologous chromosomes at each stage of oogenesis, with each chromatid and the two DNA strands per chromatids (Watson and Crick strands shown in light colors). Before EdU incorporation, each chromatid contains two unlabelled DNA strands (in light blue). Chromatids therefore appear blue. During the next S phase in the presence of EdU, the newly synthesized strains are pink. Hence, each chromatid appears pink. After chromatid segregation and a new S phase without EdU, one chromatid is entirely blue (because it has inherited the first unlabelled DNA strand and a new unlabelled DNA strand has been produced), whereas the other chromatid is pink (because although a new unlabelled DNA strand has been produced, it has inherited the labelled DNA strand from the previous cell cycle). The duration of the EdU exposure and progression through meiosis without EdU has been optimized to obtain such pattern. B) The analysis of chromosome colors at diakinesis strongly suggest that in fact, after the first S phase with EdU, the cells undergo two rounds of S phase in the absence of EdU.

Fig. S3

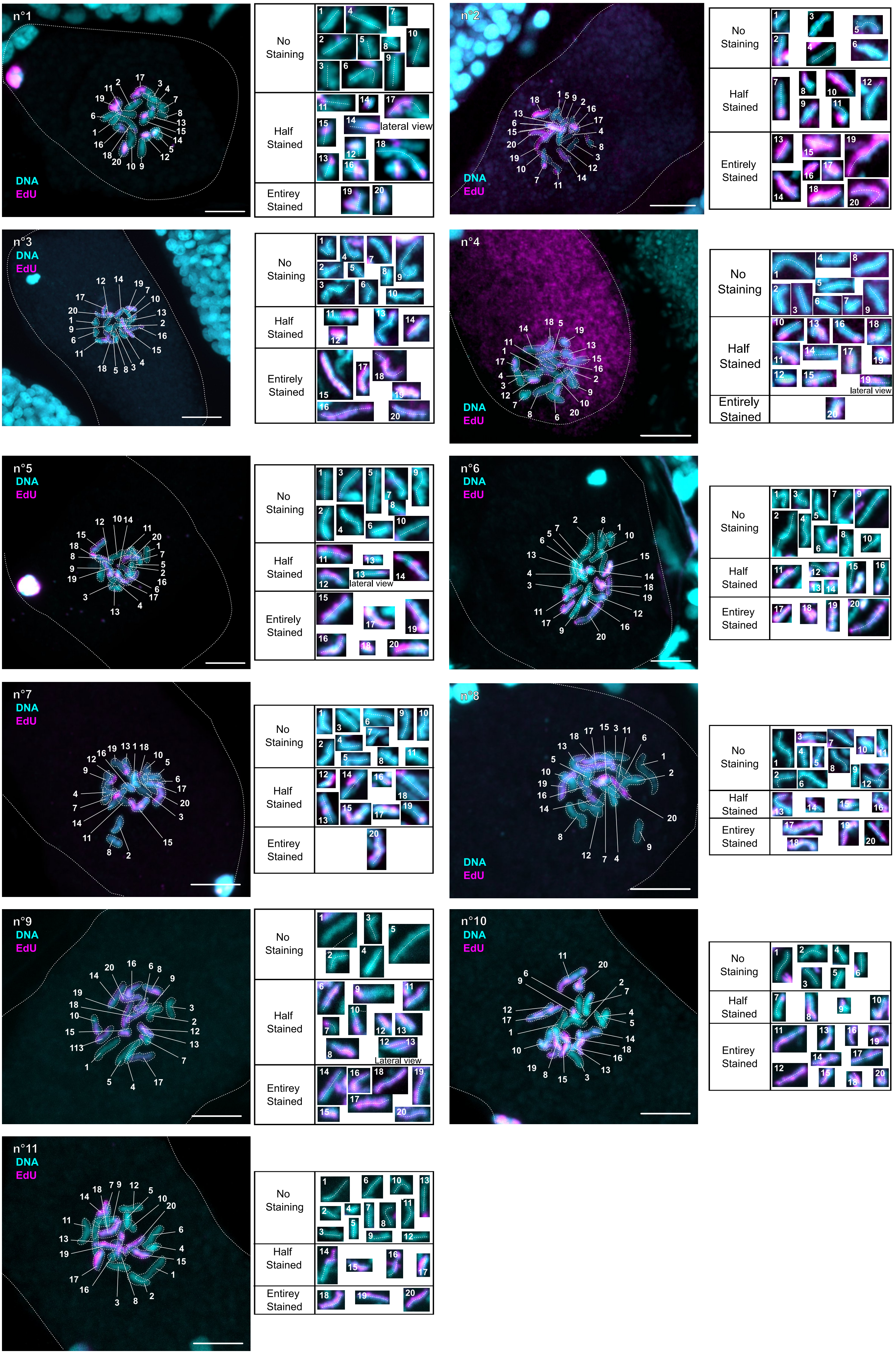
n°1
DNA
EdU
No Staining
Half Stained
lateral view
Entirely Stained
n°2
DNA
EdU
No Staining
Half Stained
Entirely Stained
n°3
DNA
EdU
No Staining
Half Stained
Entirely Stained
n°4
DNA
EdU
No Staining
Half Stained
lateral view
Entirely Stained
n°5
DNA
EdU
No Staining
Half Stained
lateral view
Entirely Stained
n°6
DNA
EdU
No Staining
Half Stained
Entirey Stained
n°7
DNA
EdU
No Staining
Half Stained
Entirey Stained
n°8
DNA
EdU
No Staining
Half Stained
Entirely Stained
n°9
DNA
EdU
No Staining
Half Stained
Lateral view
Entirey Stained
n°10
DNA
EdU
No Staining
Half Stained
Entirely Stained
n°11
DNA
EdU
No Staining
Half Stained
Entirey Stained

Figure S3: **Evidence of Directed Chromatid Assortment**.
11 images of fixed embryos at one-cell stage. EdU is in pink and DNA is in blue. The count of bicolored chromosomes is summarized in Figure 3. The dotted line shows the chromosome longest axis. Scale bar is 5 µm. Some chromosomes are shown twice, on the transverse and lateral view to better show the chromatid axis. The polar body (with labelled EdU chromatids, as expected) is visible in embryos #1 and #5. The sperm DNA (unlabeled) is visible in embryos # 3, #6 and #7.

Strain	Country	Location	Heterozygosity
JU2856	France	Angles-sur-l'Anglin. Indre	0.01381808
JU2859	United Kingdom	Cambridge	0.01531046
JU2817	France	Orsay	0.00735625
JU3129	United Kingdom	Coventry	0.01419700
JU3151	France	Saint-Aigny. Indre	0.01280673
JU3152	France	Saint-Aigny. Indre	0.01240229
JU3157	Germany	Heidelberg	0.01400762
JU3158	Germany	Heidelberg	0.01366134
JU3159	Germany	Postdam	0.01459582
JU3388	Ireland	Inis Mor. Aran Islands	0.01361719

Table S1: Measure of heterozygosity in 10 strains of *M. belari*

Supplementary Text

Population genomics and evolution of reproduction in *Mesorhabditis*

August 15, 2023

Contents

1 Population genetic structure under the *Mesorhabditis* life cycle **2**

1.1 Model . . . 2

1.1.1 General presentation and definition of parameters . . . 2

1.1.2 Recursions . . . 4

1.1.3 Simulations . . . 7

1.2 Results . . . 8

1.2.1 F_{IS} . . . 8

1.2.2 Linkage disequilibrium . . . 10

1.2.3 Conclusion . . . 11

2 Evolution of the reproductive system **13**

2.1 Genotype to phenotype map . . . 13

2.2 Inbreeding depression . . . 14

2.3 Extinction-recolonisation cycle of subpopulations . . . 14

1 Population genetic structure under the *Mesorhabditis* life cycle

1.1 Model

1.1.1 General presentation and definition of parameters

The aim of the model is to predict population genomic patterns expected under the *Mesorhabditis* life cycle. We consider a single neutral locus with an infinite allele model (IAM) of mutation. We consider a subdivided population with K demes, each of size N. The total population is thus $N_T = KN$. The life cycle is as follows (Figure S4 redrawn from the main text). The generations are discrete and non-overlapping. Migration occurs before reproduction according to the island model at rate m. This simple population structure allows modelling the breeding structure of *Mesorhabditis* with high level of consanguineous mating: the lower the deme size and the migration rates, the higher the level of consanguineous mating.

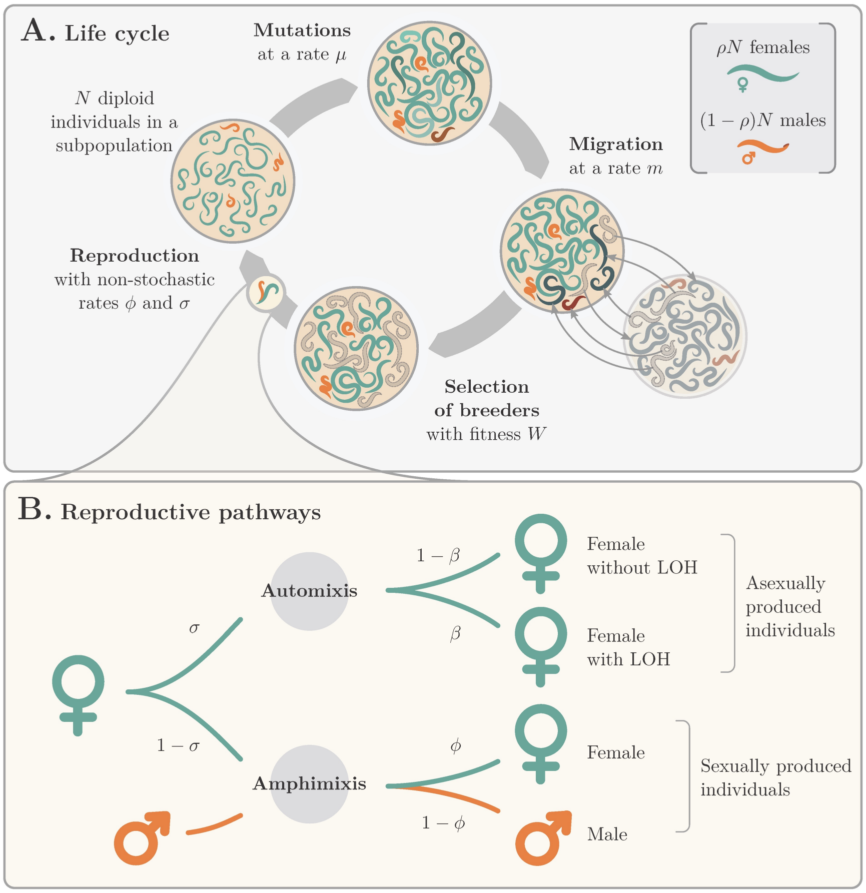

Figure S4: *Mesorhabditis* life cycle and parameters of the model.

Automictic reproduction that leads to gynogenetic females occurs in proportion σ and sexual reproduction $1-\sigma$. Under automixis, the rate of loss of heterozygosity (LOH) is β and depends on the underlying mechanism. With standard central fusion $\beta = 1/2$ after one crossover and random assortment of chromatids, and $\beta = 0$ if there is no recombination. In the proposed model of co-segregation of recombinant chromatids (CRC), $\beta = \frac{1}{2}(1-b)$, where b represents the segregation bias. If $b = 0$, there is no bias so that $\beta = 1/2$ as for recombinant chromatids under standard central fusion. If $b = 1$, the segregation is fully biased towards the co-segregation of the two recombinant chromatids. In this model, negative b values could also be considered, with $b = -1$ corresponding to complete LOH after one meiosis. However, we did not explore this possibility that is not relevant for the *Mesorhabditis* system. Under sexual reproduction, the proportion of females and males is ϕ and $1-\phi$, respectively. So far, no sexually-produced female has been observed (corresponding to ϕ=0). However, it is possible that they are produced at low rate in natural populations. If we set $\sigma = 1$ and $\beta = 0$, this is equivalent to a fully clonal model. If we set $\sigma = 0$ and $\phi = 1/2$, this is equivalent to a fully sexual model.

To obtain measures of genetic diversity and population structure (F-statistics) we derive recursions on a set of probabilities of identity by descent (IBD), Q_i, which leads to heterozygozity and F-statistics measure of the form [51]:

$$H_i = 1 - Q_i \tag{1}$$

and

$$F_{i,j} = \frac{Q_i - Q_j}{1 - Q_j} \tag{2}$$

To fully describe the model, we need to follow eight probabilities of IBD, noted Q_0^k when the two genes are sampled in the same individual, Q_1^k when they are sampled in two individuals of the same population, and Q_2^k, when they are sampled in two individuals from different populations. The superscript k stands for the sex of sampled individuals (see Figure S5).

From the parameters describing reproductive pathways (Figure S4) we obtain the proportion of males, sexual and gynogenetic females as:

$$\rho = (1-\sigma)(1-\phi) \tag{3a}$$

$$x_f = (1-\sigma)\phi \tag{3b}$$

$$x_g = \sigma \tag{3c}$$

from which we obtain the number of males and females in the populations and the proportions

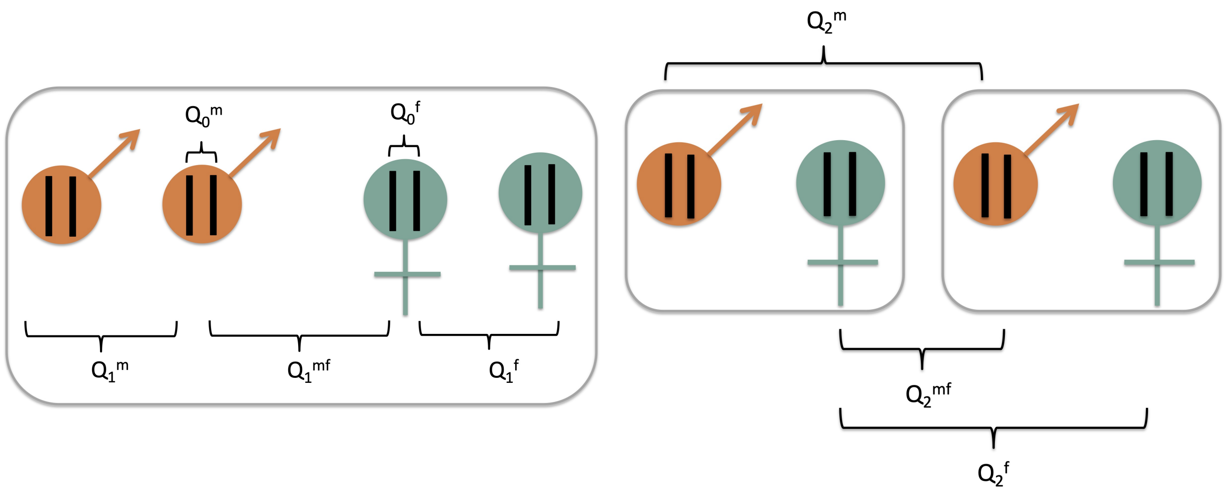

Figure S5: Definition of the eight probabilities of IBD. The grey boxes corresponds to demes.

of gynogenetic females among females

$$N_m = \rho N \tag{4a}$$
$$N_f = (1-\rho)N \tag{4b}$$
$$R_g = \frac{x_g}{x_g + x_f} \tag{4c}$$
$$\tag{4d}$$

We also need to introduce the following compound parameters:

$$\Upsilon = (1-u)^2 \tag{5a}$$
$$a = (1-m)^2 + \frac{m^2}{K-1} \tag{5b}$$
$$c = \frac{1-a}{K-1} \tag{5c}$$

Υ corresponds to the probability that the two sampled genes have not mutated in one generation, a, respectively b, corresponds to the probabilities that two individuals sampled in a same population, respectively in two different populations, were in the same population before migration.

1.1.2 Recursions

We now write the recursions of the $Q_i(t+1)$ as a function of the $Q_i(t)$. At equilibrium $Q_i(t+1) = Q_i(t)$ so we remove the time subscript and directly give the equations at

equilibrium:

$$
\begin{aligned}
Q_0^f =& \Upsilon\big(R_g\big(\beta+(1-\beta)Q_0^f\big)+(1-R_g)Q_1^{mf}\big) && \text{(6a)}\\
Q_0^m =& \Upsilon Q_1^{mf} && \text{(6b)}\\
Q_1^f =& \Upsilon\Bigg(a\Bigg(R_g^2\bigg(\frac{1}{N_f}(\frac{1+Q_0^f}{2})+\big(1-\frac{1}{N_f}\big)Q_1^f\bigg)\\
&+2R_g(1-R_g)\bigg(\frac{1}{2}(\frac{1}{N_f}(\frac{1+Q_0^f}{2})+\big(1-\frac{1}{N_f}\big)Q_1^f)+\frac{Q_1^{mf}}{2}\bigg)\\
&+(1-R_g)^2\bigg(\frac{1}{4}(\frac{1}{N_f}(\frac{1+Q_0^f}{2})+\big(1-\frac{1}{N_f}\big)Q_1^f)+\frac{1}{4}(\frac{1}{N_m}(\frac{1+Q_0^m}{2})+\big(1-\frac{1}{N_m}\big)Q_1^m)+\frac{1}{2}Q_1^{mf}\bigg)\Bigg)\\
&+\big(1-a\big)\bigg(R_g^2Q_2^f+R_g(1-R_g)(Q_2^f+Q_2^{mf})+(1-R_g)^2(\frac{Q_2^f}{4}+\frac{Q_2^m}{4}+\frac{Q_2^{mf}}{2})\bigg)\Bigg) && \text{(6c)}\\
Q_1^m =& \Upsilon\Bigg(a\bigg(\frac{1}{4}(\frac{1}{N_f}(\frac{1+Q_0^f}{2})+\big(1-\frac{1}{N_f}\big)Q_1^f)+\frac{1}{4}(\frac{1}{N_m}(\frac{1+Q_0^m}{2})+\big(1-\frac{1}{N_m}\big)Q_1^m)+\frac{1}{2}Q_1^{mf})\bigg)\\
&+\big(1-a\big)(\frac{Q_2^f}{4}+\frac{Q_2^m}{4}+\frac{Q_2^{mf}}{2})\Bigg) && \text{(6d)}\\
Q_1^{mf} =& \Upsilon\Bigg(a\Bigg(R_g\bigg(\frac{1}{2}(\frac{1}{N_f}(\frac{1+Q_0^f}{2})+\big(1-\frac{1}{N_f}\big)Q_1^f)+\frac{Q_1^{mf}}{2}\bigg)\\
&+(1-R_g)\bigg(\frac{1}{4}(\frac{1}{N_f}(\frac{1+Q_0^f}{2})+\big(1-\frac{1}{N_f}\big)Q_1^f)+\frac{1}{4}(\frac{1}{N_m}(\frac{1+Q_0^m}{2})+\big(1-\frac{1}{N_m}\big)Q_1^m)+\frac{Q_1^{mf}}{2}\bigg)\Bigg)\\
&+\big(1-a\big)\bigg(R_g(\frac{Q_2^f}{2}+\frac{Q_2^{mf}}{2})+(1-R_g)(\frac{Q_2^f}{4}+\frac{Q_2^m}{4}+\frac{Q_2^{mf}}{2})\bigg)\Bigg) && \text{(6e)}\\
Q_2^f =& \Upsilon\Bigg(c\Bigg(R_g^2\bigg(\frac{1}{N_f}(\frac{1+Q_0^f}{2})+\big(1-\frac{1}{N_f}\big)Q_1^f\bigg)\\
&+2R_g(1-R_g)\bigg(\frac{1}{2}(\frac{1}{N_f}(\frac{1+Q_0^f}{2})+\big(1-\frac{1}{N_f}\big)Q_1^f)+\frac{Q_1^{mf}}{2}\bigg)\\
&+(1-R_g)^2\bigg(\frac{1}{4}(\frac{1}{N_f}(\frac{1+Q_0^f}{2})+\big(1-\frac{1}{N_f}\big)Q_1^f)\\
&+\frac{1}{4}(\frac{1}{N_m}(\frac{1+Q_0^m}{2})+\big(1-\frac{1}{N_m}\big)Q_1^m)+\frac{Q_1^{mf}}{2})\bigg)\Bigg)\\
&+\big(1-c\big)\bigg(R_g^2Q_2^f+R_g(1-R_g)(Q_2^f+Q_2^{mf})+(1-R_g)^2(\frac{Q_2^f}{4}+\frac{Q_2^m}{4}+\frac{Q_2^{mf}}{2})\bigg)\Bigg) && \text{(6f)}\\
Q_2^m =& \Upsilon\Bigg(c\bigg(\frac{1}{4}(\frac{1}{N_f}(\frac{1+Q_0^f}{2})+\big(1-\frac{1}{N_f}\big)Q_1^f)\\
&+\frac{1}{4}(\frac{1}{N_m}(\frac{1+Q_0^m}{2})+\big(1-\frac{1}{N_m}\big)Q_1^m+\frac{Q_1^{mf}}{2}\bigg)+\big(1-c\big)(\frac{Q_2^f}{4}+\frac{Q_2^m}{4}+\frac{Q_2^{mf}}{2})\Bigg) && \text{(6g)}
\end{aligned}
$$

(continued on next page)

(continued from previous page)

$$Q_2^{mf} = \Upsilon\Bigg(c\Bigg(R_g\Bigg(\frac{1}{2}(\frac{1}{N_f}(\frac{1+Q_0^f}{2}) + (1-\frac{1}{N_f})Q_1^f) + \frac{Q_1^{mf}}{2}\Bigg)$$
$$+ (1-R_g)\Bigg(\frac{1}{4}(\frac{1}{N_f}(\frac{1+Q_0^f}{2}) + (1-\frac{1}{N_f})Q_1^f) + \frac{1}{4}(\frac{1}{N_m}(\frac{1+Q_0^m}{2}) + (1-\frac{1}{N_m})Q_1^m) + \frac{Q_1^{mf}}{2}\Bigg)\Bigg)$$
$$+ (1-c)\Bigg(R_g(\frac{Q_2^f}{2} + \frac{Q_2^{mf}}{2}) + (1-R_g)(\frac{Q_2^f}{4} + \frac{Q_2^m}{4} + \frac{Q_2^{mf}}{2})\Bigg)\Bigg) \tag{6d}$$

As an example, the rationale of the derivation is given for $Q_0^f(t+1)$. The two copies sampled in a female are IBD first if none has mutated (Υ). Then we must consider that this female is gynogenetic (R_g) or sexual ($1-R_g$). If it is gynogenetic, if heterozygozity has been lost (β) the two copies are IBD with probability one, otherwise $(1-\beta)$ the probability of IBD is the same as for the mother, so $Q_0^f(t)$. If the female come from sexual reproduction, the two copies are IBD with the same probability as of a random male/female pair at the previous generation, Q_1^{mf}. The terms in $1/N_m$ and $1/N_f$ that appear in equations for the Q_1^k and Q_2^k correspond to the probability that two different individuals have the same father or mother, respectively.

This system of recursions can be written in the matrix form:

$$\vec{Q} = \Upsilon(\mathbf{G}\vec{Q} + \vec{C}) \tag{7}$$

where $\vec{Q}$ is the vector of probabilities of IBD,$\mathbf{G}$ is a matrix and $\vec{C}$ a vector, both depending of the parameters of the model. The solution can be written on the form:

$$\vec{Q} = (\mathbf{I} - \Upsilon\mathbf{G})^{-1}\Upsilon\vec{C} \tag{8}$$

where $\mathbf{I}$ is the identity matrix.

The F_{IS} statistics measured on genomic data corresponds to $F_{0,2}$ in our model as we compare the IBD of two gene copies sampled either within an individual or at random over the whole population. It can be defined either for males, females, or for the whole population

by weighting as a function of the sex-ratio:

$$F_{IS}^m = \frac{Q_0^m - Q_2^m}{1 - Q_2^m} \tag{9a}$$

$$F_{IS}^f = \frac{Q_0^f - Q_2^f}{1 - Q_2^f} \tag{9b}$$

$$F_{IS} = x_m \frac{Q_0^m - x_m Q_2^m - (1 - x_m) Q_2^f}{1 - x_m Q_2^m - (1 - x_m) Q_2^f} + (1 - x_m) \frac{Q_0^f - x_m Q_2^m - (1 - x_m) Q_2^f}{1 - x_m Q_2^m - (1 - x_m) Q_2^f} \tag{9c}$$

1.1.3 Simulations

In addition to analytical derivations, an individual-based, multi-locus model was implemented using the SLiM software [50], where each individual's genome is explicitly defined. It allowed to simulate genomic patterns along a chromosome and to introduce deleterious mutations in the model. Each individual is represented by a unique pair of autosome. This chromosome consist of $L = 10^5$ loci, with a rate of recombination per locus r equal to $1/L = 10^{-5}$, so that on average one recombination event is observed per gametogenesis. Since *M. belari* has holocentric chromosomes (there is no specific centromere), the location of the chiasma is randomly drawn according to a uniform distribution along the chromosome. Two types of mutations are introduced, either neutral (no effect on fitness) or deleterious ones, with selection coefficient s, set to 0.01, and dominance coefficient h, set to 0.25, and acting multiplicatively across the genome. The life cycle was the same as described above. Various population sizes and migration rates were explored to modulate the level of inbreeding and we specifically studied the effect of the rate of production of sexual females, ϕ and the rate of LOH, β.

By adjusting the parameters of the model, we also compared the *M. belari* life cycle with more standard reproductive modes: full sexuality ($\sigma = 0$ and $\phi = 1/2$), full clonality ($\sigma = 1$ and $r = 0$), and central fusion automixis ($\sigma = 1$, and $\beta = 1/2$).

From the simulations we computed F_{IS} and the linkage-disequilibrium measure r^2 on neutral mutations at the scale of the whole population:

$$\mathrm{F_{IS}} = 1 - \frac{H_o}{H_e} \tag{10}$$

with H_o the observed heterozygosity (percentage of heterozygous sites) and H_e the expected heterozygosity :

$$H_e = \frac{2}{L} \sum_{i=1}^{n} f_i (1 - f_i) \tag{11}$$

a sum over all the n mutations present in the population with f_i their respective frequencies.

$$r^2 = \frac{D^2}{\pi_A \pi_a \pi_B \pi_b} \tag{12}$$

where $D = \pi_{AB} - \pi_A \pi_B$ with π_A and π_a the allelic frequency at a first locus A and π_B and π_b at a second locus B. Only mutations in frequency higher than 5% in the population were used. r^2 was calculated for pairs of loci as the function of their distance on the simulated chromosome.

1.2 Results

1.2.1 F_{IS}

The general solution of equation (8) can be obtained with the help of *Mathematica* [52] but it is formidable, so useless for direct biological interpretation. We thus performed numerical explorations in the general case. We also obtained approximations under two limit conditions: $\phi = 0$ (no sexually-produced females) and $\beta = 0$ (no LOH). Under these two conditions we also used the standard diffusion limit. First we used the following scaling parameters: $\theta = 4N_T u$, $\Phi = 2N_T \phi$, $B = 2N_T \beta$. Then, we assumed an infinite number of local populations so $K, N_T \to \infty$ but that the scaled parameters terms tends towards constant: $\theta, \Phi, B \to cte$. We also noted $M = 4Nm$, where migration is scaled with the local, N, which can be small.

Assuming no sexually-produced females we obtained:

$$F_{IS}^m \approx \frac{2B + \theta}{2(B + \theta) + M(\sigma B + \theta\sigma + 1)} \tag{13a}$$

$$F_{IS}^f \approx \frac{2B + M(\sigma B - 1)}{2(B + \theta) + M(\sigma B + \theta\sigma + 1)} \tag{13b}$$

$$F_{IS} \approx \frac{2B + \theta(1 - \sigma) + \sigma M(\sigma B - 1)}{2(B + \theta) + M(\sigma B + \theta\sigma + 1)} \tag{13c}$$

Assuming no LOH we obtained:

$$F_{IS}^m \approx \frac{(1 - \sigma)\Phi + \sigma\theta}{(1 - \sigma)\Phi(1 + M\sigma) + \sigma(2\theta + M(1 + \sigma\theta))} \tag{14a}$$

$$F_{IS}^f \approx \frac{(1 - \sigma)\Phi - \sigma M}{(1 - \sigma)\Phi(1 + M\sigma) + \sigma(2\theta + M(1 + \sigma\theta))} \tag{14b}$$

$$F_{IS} \approx \frac{(1 - \sigma)(\sigma\theta + \Phi) - M\sigma^2}{(1 - \sigma)\Phi(1 + M\sigma) + \sigma(2\theta + M(1 + \sigma\theta))} \tag{14c}$$

We give the three expressions for completeness but we can concentrate on the expression for the average F_{IS} In natural populations, there is a strong family structure with most

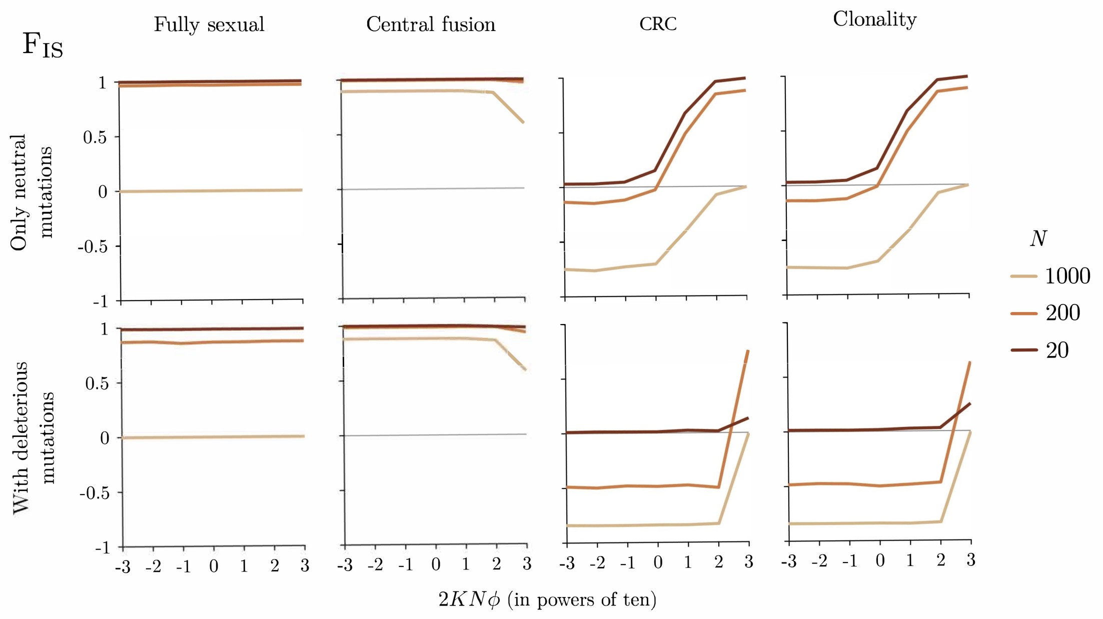

Figure S6: F_{IS} for the different reproductive modes as a function of the rate of sexually produced females ($2KN\phi$) for different levels of population structure ($KN = 1000$, so $N = 1000$ corresponds to a single populations and $N = 20$ to a highly structured one).

matings occurring between kins. In our model, it corresponds to low migration between demes, so M close to 0. Then, equations (13c) and (14c) become:

$$F_{IS} \approx \frac{2B + \theta(1-\sigma)}{2(B+\theta)} \tag{15}$$

$$F_{IS} \approx \frac{(1-\sigma)(\sigma\theta + \Phi)}{(1-\sigma)\Phi + 2\sigma\theta} \tag{16}$$

which both reduce to $F_{IS} = (1-\sigma)/2$ when $B = 0$ or $\Phi = 0$. As $\sigma \approx 0.9$ in natural populations it corresponds to $F_{IS} \approx 0.05$, so close to the observed value ($F_{IS} \approx 0.19$, see main text). In an unstructured population ($M \to \infty$, and see $N = 1000$ on Figure S6), F_{IS} tends to -1 with no LOH ($B = 0$) (see also [48]). Here, this is compensated by the strong family structure leading to F^f_{IS} close to 0 as illustrated on Figure S6 (see also [?]). It is worth noting that just a little bit of sex or LOH (higher than the mutation rate: $\phi, B > \theta$) rapidly leads to F^f_{IS} close to 1. This is confirmed by simulations as presented (Figure S6 and Figure

4 in the main text). This result is thus very sensitive to the occurrence of sexually produced females and to low level of LOH. For comparison, we also present the case of standard central fusion, clonality and fully sexual reproduction.

However, simulations showed that because of deleterious mutations, the range of parameters leading to F_{IS}^f close to 0 can be much wider, even when some females are sexually produced and when the CRC is not complete (Figure S7). This is explained by the selection against highly homozygous individuals. This is an important result as a non-zero proportion of sexually produced females is required to explain the low level of linkage disequilibrium observed genome wide as presented below.

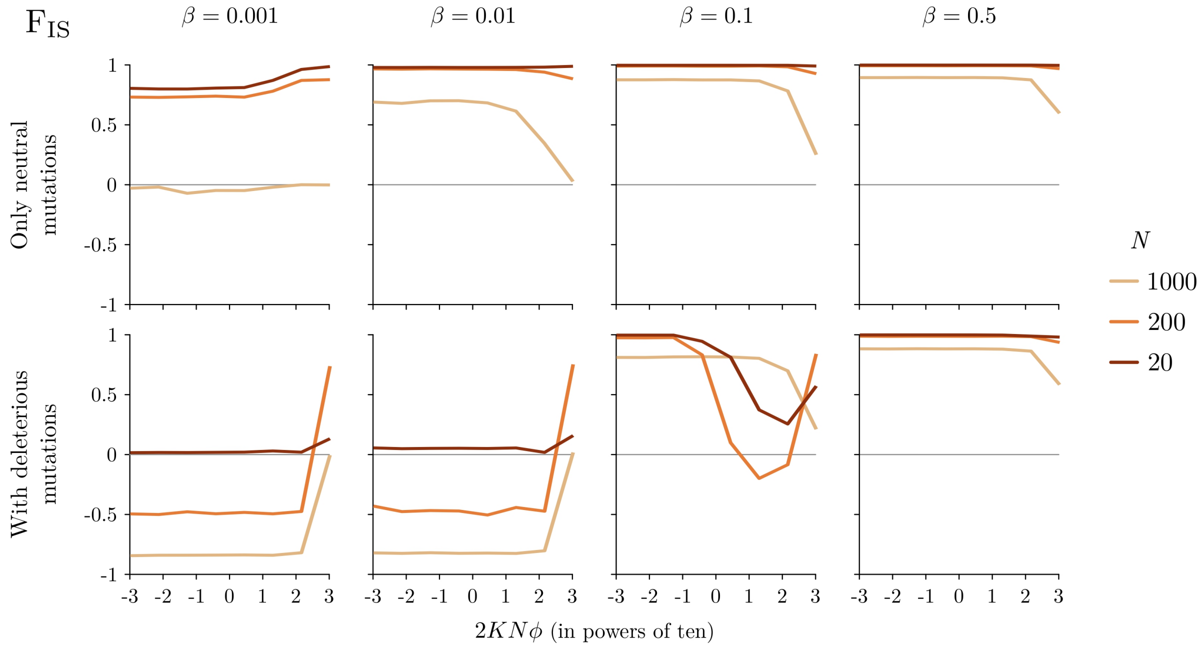

Figure S7: F_{IS} for automixis with different level of CRC ($\beta = 0$ corresponds to full co-segregation and $\beta = 1/2$ to random pairing).

For comparison, Figures S6 and S7 also shows the results for full sexuality, full clonality and standard central fusion automixis (without CRC).

1.2.2 Linkage disequilibrium

Analytical derivations for linkage disequilibrium would require recursion equations for 40 IBD probabilities. We thus only relied on simulations.

To understand the specific effect of the CRC we first considered a single unstructured population. As expected, LD is low and rapidly decreases with physical distance under sexual reproduction (Figure S8). In contrast, LD is high and independent of physical distance along chromosome under both full clonality because recombination does not occur, and

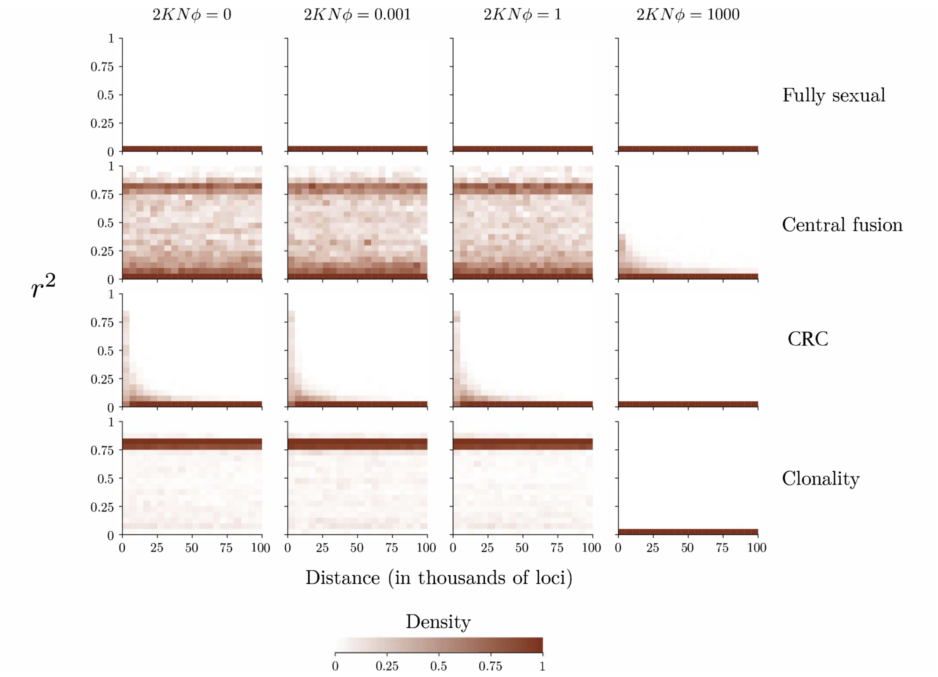

Figure S8: Pattern of linkage disequilibrium as a function of physical distance (in number of loci) for different rates of sexually produced females ($2NK\phi = 0.001, 1, 100$) and for different reproductive modes.

under standard central fusion because recombination is not efficient as individuals are mostly homozygotes. Under complete CRC and without sexually produced females, LD pattern is intermediate: much lower than under clonality or standard central fusion but higher than under full sexuality, and decreasing with physical distance (Figure S8). The reason is that, although new haplotypes are generated by recombination within individuals, they are never associated together through mating, so recombination is not as efficient as under full sexuality.

When we also consider the effect of population structure, LD is globally higher and the difference among reproductive modes are less clear under pure neutrality. However, when deleterious mutations are added the differences become stronger. In particular a very low rate of sexually produced females is sufficient to make CRC similar to full sexuality whereas much higher rates are necessary to erase the signature of clonality or standard central fusion (Figure S9).

1.2.3 Conclusion

Overall, the observed genomic pattern is well predicted by the strong family structure of *M. belari* populations directed chromatif assortment, sexually produced females at low rate (which can be unobserved in natural conditions), and the occurrence of deleterious mutations

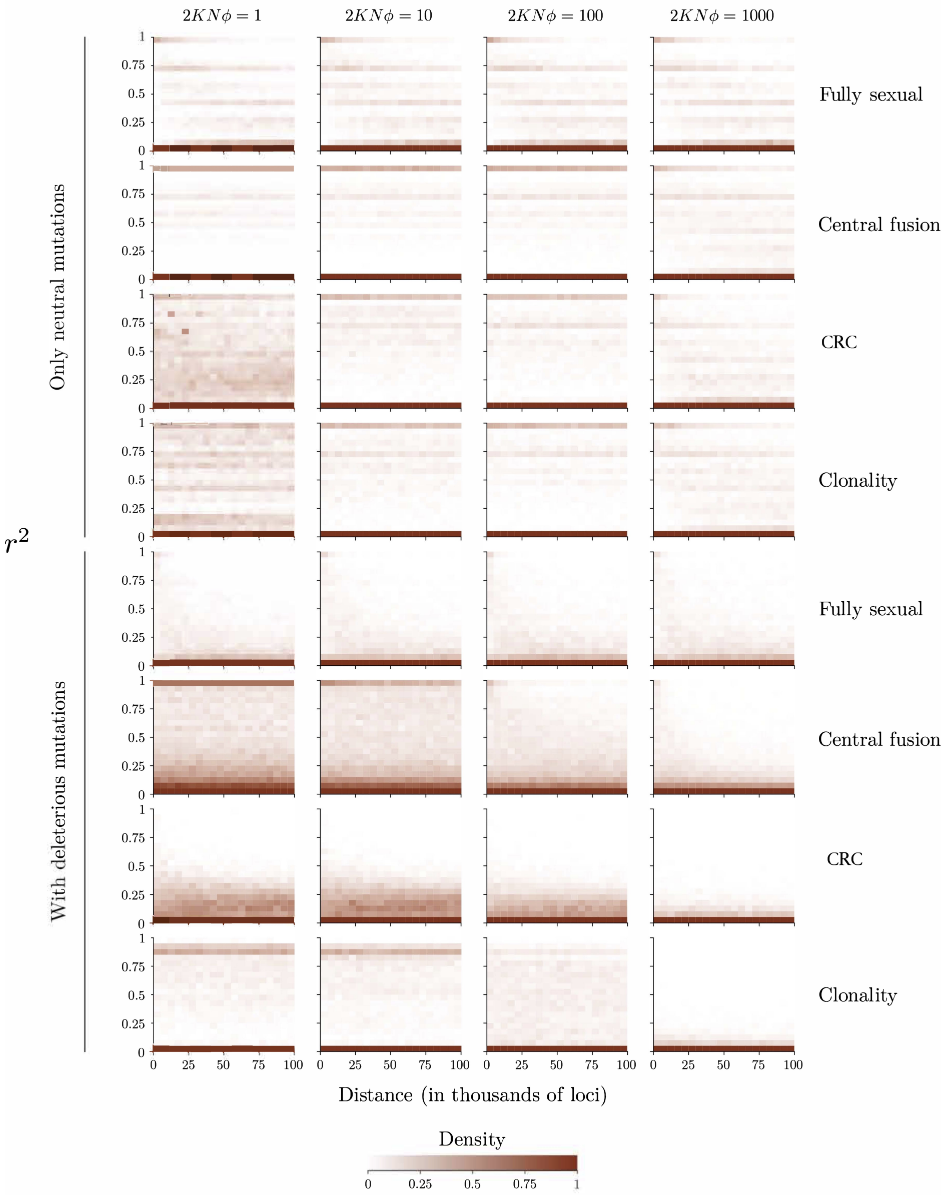

Figure S9: Pattern of linkage disequilibrium as a function of physical distance (in number of loci) for different rates of sexually produced females ($2NK\phi = 0.001, 1, 100$) and for different reproductive modes.

genome wide.

2 Evolution of the reproductive system

In the second model, we study the evolution of the atypical reproductive system of *M. belari* from a standard sexual population. Compared to the previous model, the parameters σ (proportion of asexually produced offspring) and β (rate of LOH during automixis) are no longer fixed but under the control of two independent evolving loci. Among sexually produced individuals the proportion of females is set to $\phi = 1/2$. Additional simulations where the sex-ratio can also evolve do not change the results and a sex ratio biased towards males evolved in a second step (not shown).

We started with a burn-in period where the population evolved under sexual reproduction. Then mutations are introduced at one or at the two loci controlling the reproductive mode. Each evolving locus is unlinked with any other locus. Transmission from parents to children of an evolving locus follows the same rules as transmission of loci on the focal chromosome, taking into account the potential LOH through chromosome segregation during automixis. It can take two different values, a resident value, g_r, or a mutant value, $g_m = g_r \pm \delta$. We used $\delta = 0.1$ to avoid too long simulations. We assumed additivity between the two alleles so the genotypic value is the sum of the two alleles. At the beginning of simulations, all individuals are homozygous for the resident allele and a mutant allele is randomly introduced in one individual of the population. If this mutant becomes fixed in the population, this mutant allele becomes the resident allele. If the mutant disappears, another mutant is directly reintroduced into the population at random.

2.1 Genotype to phenotype map

The value of σ and β are constrained between 0 and 1. The simulations start with a sexual population, with a rate of pseudogamy set to 0 and that can theoretically go up to 1. The rate of LOH β starts at 1/2 and can reach 0 (no LOH, complete cosegregation of recombinant chromatids) or 1 (full LOH, complete segregation of one recombinant and one non-recombinant chromatids). To map allelic values from $]-\infty, +\infty[$ to $[0, 1]$ we used the following functions:

$$P_\sigma = f(G_\sigma) \quad ; \quad P_\beta = \frac{1}{2}\left(1 - f(G_\beta)\right) \tag{17}$$

with

$$f(x) = \begin{cases} 1 - \left(1 - \frac{2}{\pi}\tan^{-1}(ax)\right)^b, & \text{if } g > 0 \\ \left(1 - \frac{2}{\pi}\tan^{-1}(-ax)\right)^b - 1, & \text{otherwise} \end{cases} \tag{18}$$

The parameters a and b define whether the function tends more or less quickly to 1 as x increases. We used $a = 0.1$ and $b = 15$ but equilibrium results are not sensitive to chosen

values. The rate of pseudogamy and the rate of LOH are controlled by the maternal genotype.

2.2 Inbreeding depression

The supposedly strong barrier to the evolution of asexuality (assuming that automixis mechanisms can emerge) is inbreeding depression (ID) due to LOH that leads to the exposure of recessive deleterious alleles. ID is calculated as $1 - w_1/w_0$ with w_0 being the fitness of sexually produced individuals and w_1 the fitness of asexually produced individuals.

A strong ID should hold back the evolution of automixis if the chromosome segregation bias is not strong, preventing LOH. Since we only modelled a single chromosome and central fusion leads to a LOH in only half of offsrpings, the maximum ID is 50%, which is the minimum value preventing the evolution of asexuality. Asexuality should always evolved under such a conditions. To allow a broader range of ID values we assumed an additional cost of LOH and corrected w_1 to $w_1^{corr} = (1-c)^{\beta(n-1)}w_1$, where n is the number of chromosomes (fixed to 10) and c is the mean fitness decline caused by LOH per chromosome.

2.3 Extinction-recolonisation cycle of subpopulations

In this model, a higher migration rate is set, allowing the evolving loci to be transmitted not too slowly from one population to another. It should also be noted that here the parameters controlling reproduction are no longer fixed rates. It is thus possible that populations may stochastically run out of males, especially if pseudogamy evolves and the percentage of males decreases. If a population has no more males, it becomes extinct. An extinct population is recolonised in the generation following its extinction by a male and at least one female randomly selected from the other population. This pair reproduces and together they form the next generation of the subpopulation.

REFERENCES AND NOTES

1. E. Suomalainen, A. Saura, J. Lokki, *Cytology and Evolution in Parthenogenesis* (CRC Press, 1987).

2. M. Neiman, T. F. Sharbel, T. Schwander, Genetic causes of transitions from sexual reproduction to asexuality in plants and animals. *J. Evol. Biol.* **27**, 1346–1359 (2014).

3. T. Lenormand, J. Engelstädter, S. E. Johnston, E. Wijnker, C. R. Haag, Evolutionary mysteries in meiosis. *Philos. Trans. R. Soc. Lond. B Biol. Sci.* **371**, 20160001 (2016).

4. M. Archetti, Complementation, genetic conflict, and the evolution of sex and recombination. *J. Hered.* **101**, S21–S33 (2010).

5. J. Engelstädter, Constraints on the evolution of asexual reproduction. *Bioessays* **30**, 1138–1150 (2008).

6. C. R. Haag, L. Theodosiou, R. Zahab, T. Lenormand, Low recombination rates in sexual species and sex-asex transitions. *Philos. Trans. R. Soc. Lond. B Biol. Sci.* **372**, 20160461 (2017).

7. K. S. Jaron, J. Bast, R. W. Nowell, T. R. Ranallo-Benavidez, M. Robinson-Rechavi, T. Schwander, Genomic features of parthenogenetic animals. *J. Hered.* **112**, 19–33 (2021).

8. A. Brandt, P. Tran Van, C. Bluhm, Y. Anselmetti, Z. Dumas, E. Figuet, C. M. François, N. Galtier, B. Heimburger, K. S. Jaron, M. Labédan, M. Maraun, D. J. Parker, M. Robinson-Rechavi, I. Schaefer, P. Simion, S. Scheu, T. Schwander, J. Bast, Haplotype divergence supports long-term asexuality in the oribatid mite *Oppiella nova*. *Proc. Natl. Acad. Sci. U.S.A.* **118**, e2101485118 (2021).

9. P. Simion, J. Narayan, A. Houtain, A. Derzelle, L. Baudry, E. Nicolas, R. Arora, M. Cariou, C. Cruaud, F. R. Gaudray, C. Gilbert, N. Guiglielmoni, B. Hespeels, D. K. L. Kozlowski, K. Labadie, A. Limasset, M. Llirós, M. Marbouty, M. Terwagne, J. Virgo, R. Cordaux, E. G. J. Danchin, B. Hallet, R. Koszul, T. Lenormand, J.-F. Flot, K. Van Doninck, Chromosome-level genome assembly reveals homologous chromosomes and recombination in asexual rotifer *Adineta vaga*. *Sci. Adv.* **7**, eabg4216 (2021).

10. P. Tran Van, Y. Anselmetti, J. Bast, Z. Dumas, N. Galtier, K. S. Jaron, K. Martens, D. J. Parker, M. Robinson-Rechavi, T. Schwander, P. Simion, I. Schön, First annotated draft genomes of nonmarine ostracods (Ostracoda, Crustacea) with different reproductive modes. *G3 (Bethesda)* **11**, jkab043 (2021).

11. H. Fradin, K. Kiontke, C. Zegar, M. Gutwein, J. Lucas, M. Kovtun, D. L. Corcoran, L. R. Baugh, D. H. A. Fitch, F. Piano, K. C. Gunsalus, Genome architecture and evolution of a unichromosomal asexual nematode. *Curr. Biol.* **27**, 2928–2939.e6 (2017).

12. R. Guidetti, M. Cesari, R. Bertolani, T. Altiero, L. Rebecchi, High diversity in species, reproductive modes and distribution within the *Paramacrobiotus richtersi* complex (Eutardigrada, Macrobiotidae). *Zool. Lett.* **5**, 1 (2019).

13. M. Grosmaire, C. Launay, M. Siegwald, T. Brugière, L. Estrada-Virrueta, D. Berger, C. Burny, L. Modolo, M. Blaxter, P. Meister, M.-A. Félix, P.-H. Gouyon, M. Delattre, Males as somatic investment in a parthenogenetic nematode. *Science* **363**, 1210–1213 (2019).

14. D. G. Albertson, J. N. Thomson, Segregation of holocentric chromosomes at meiosis in the nematode, *Caenorhabditis elegans*. *Chromosome Res.* **1**, 15–26 (1993).

15. D. E. Almanzar, S. G. Gordon, O. Rog, Meiotic sister chromatid exchanges are rare in *C. elegans*. *Curr. Biol.* **31**, 1499–1507.e3 (2021).

16. A.-C. Valfort, C. Launay, M. Sémon, M. Delattre, Evolution of mitotic spindle behavior during the first asymmetric embryonic division of nematodes. *PLOS Biol.* **16**, e2005099 (2018).

17. C. Launay, M.-A. Félix, J. Dieng, M. Delattre, Diversification and hybrid incompatibility in auto-pseudogamous species of *Mesorhabditis* nematodes. *BMC Evol. Biol.* **20**, 105 (2020).

18. T. S. Korneliussen, A. Albrechtsen, R. Nielsen, ANGSD: Analysis of next generation sequencing data. *BMC Bioinformatics* **15**, 356 (2014).

19. E. C. Andersen, J. P. Gerke, J. A. Shapiro, J. R. Crissman, R. Ghosh, J. S. Bloom, M.-A. Félix, L. Kruglyak, Chromosome-scale selective sweeps shape *Caenorhabditis elegans* genomic diversity. *Nat. Genet.* **44**, 285–290 (2012).

20. A. H. Chan, P. A. Jenkins, Y. S. Song, Genome-wide fine-scale recombination rate variation in *Drosophila melanogaster*. *PLOS Genet.* **8**, e1003090 (2012).

21. F. Balloux, L. Lehmann, T. de Meeûs, The population genetics of clonal and partially clonal diploids. *Genetics* **164**, 1635–1644 (2003).

22. S. L. Ament-Velásquez, E. Figuet, M. Ballenghien, E. E. Zattara, J. L. Norenburg, F. A. Fernández-Álvarez, J. Bierne, N. Bierne, N. Galtier, Population genomics of sexual and asexual lineages in fissiparous ribbon worms (Lineus, Nemertea): Hybridization, polyploidy and the Meselson effect. *Mol. Ecol.* **25**, 3356–3369 (2016).

23. S. Glémin, N. Galtier, Genome evolution in outcrossing versus selfing versus asexual species. *Methods Mol. Biol.* **855**, 311–335 (2012).

24. M. Mandrioli, G. C. Manicardi, Holocentric chromosomes. *PLOS Genet.* **16**, e1008918 (2020).

25. M. P. H. Stumpf, G. A. T. McVean, Estimating recombination rates from population-genetic data. *Nat. Rev. Genet.* **4**, 959–968 (2003).

26. A. Agostinho, B. Meier, R. Sonneville, M. Jagut, A. Woglar, J. Blow, V. Jantsch, A. Gartner, Combinatorial regulation of meiotic holliday junction resolution in *C. elegans* by HIM-6 (BLM) helicase, SLX-4, and the SLX-1, MUS-81 and XPF-1 nucleases. *PLOS Genet.* **9**, e1003591 (2013).

27. N. J. O'Neil, J. S. Martin, J. L. Youds, J. D. Ward, M. I. R. Petalcorin, A. M. Rose, S. J. Boulton, Joint molecule resolution requires the redundant activities of MUS-81 and XPF-1 during *Caenorhabditis elegans* meiosis. *PLOS Genet.* **9**, e1003582 (2013).

28. Y. Hong, M. Velkova, N. Silva, M. Jagut, V. Scheidt, K. Labib, V. Jantsch, A. Gartner, The conserved LEM-3/Ankle1 nuclease is involved in the combinatorial regulation of meiotic recombination repair and chromosome segregation in *Caenorhabditis elegans*. *PLOS Genet.* **14**, e1007453 (2018).

29. Y. Liu, C. F. Nielsen, Q. Yao, I. D. Hickson, The origins and processing of ultra fine anaphase DNA bridges. *Curr. Opin. Genet. Dev.* **26**, 1–5 (2014).

30. B. P. Oldroyd, B. Yagound, M. H. Allsopp, M. J. Holmes, G. Buchmann, A. Zayed, M. Beekman, Adaptive, caste-specific changes to recombination rates in a thelytokous honeybee population. *Proc. R. Soc. B Biol. Sci.* **288**, 20210729 (2021).

31. E. Altendorfer, L. I. Láscarez-Lagunas, S. Nadarajan, I. Mathieson, M. P. Colaiácovo, Crossover position drives chromosome remodeling for accurate meiotic chromosome segregation. *Curr. Biol.* **30**, 1329–1338.e7 (2020).

32. G. Taberly, [The cytology of parthenogenesis in *Platynothrus peltifer* (Koch) (Acarien, Oribate)]. *C. R. Hebd. Seances Acad. Sci.* **247**, 1655–1657 (1958).

33. M. Archetti, Evidence from automixis with inverted meiosis for the maintenance of sex by loss of complementation. *J. Evol. Biol.* **35**, 40–50 (2022).

34. M. Schvarzstein, S. M. Wignall, A. M. Villeneuve, Coordinating cohesion, co-orientation, and congression during meiosis: Lessons from holocentric chromosomes. *Genes Dev.* **24**, 219–228 (2010).

35. M. Sarens, G. P. Copenhaver, N. De Storme, The role of chromatid interference in determining meiotic crossover patterns. *Front. Plant Sci.* **12**, 656691 (2021).

36. K. J. Beumer, S. Pimpinelli, K. G. Golic, Induced chromosomal exchange directs the segregation of recombinant chromatids in mitosis of *Drosophila*. *Genetics* **150**, 173–188 (1998).

37. C. S. Ottolini, L. Newnham, A. Capalbo, S. A. Natesan, H. A. Joshi, D. Cimadomo, D. K. Griffin, K. Sage, M. C. Summers, A. R. Thornhill, E. Housworth, A. D. Herbert, L. Rienzi, F. M. Ubaldi, A. H. Handyside, E. R. Hoffmann, Genome-wide maps of recombination and chromosome segregation in human oocytes and embryos show selection for maternal recombination rates. *Nat. Genet.* **47**, 727–735 (2015).

38. P. R. Oxley, L. Ji, I. Fetter-Pruneda, S. K. McKenzie, C. Li, H. Hu, G. Zhang, D. J. C. Kronauer, The genome of the clonal raider ant *Cerapachys biroi*. *Curr. Biol.* **24**, 451–458 (2014).

39. M. Dukić, D. Berner, C. R. Haag, D. Ebert, How clonal are clones? A quest for loss of heterozygosity during asexual reproduction in *Daphnia magna*. *J. Evol. Biol.* **32**, 619–628 (2019).

40. L. Serra, D. Z. Chang, M. Macchietto, K. Williams, R. Murad, D. Lu, A. R. Dillman, A. Mortazavi, Adapting the Smart-seq2 protocol for robust single worm RNA-seq. *Bio. Protoc.* **8**, e2729 (2018).

41. H. Li, R. Durbin, Fast and accurate short read alignment with Burrows-Wheeler transform. *Bioinformatics* **25**, 1754–1760 (2009).

42. H. Li, B. Handsaker, A. Wysoker, T. Fennell, J. Ruan, N. Homer, G. Marth, G. Abecasis, R. Durbin; 1000 Genome Project Data Processing Subgroup, The sequence alignment/map format and SAMtools. *Bioinformatics* **25**, 2078–2079 (2009).

43. A. M. Bolger, M. Lohse, B. Usadel, Trimmomatic: A flexible trimmer for Illumina sequence data. *Bioinformatics* **30**, 2114–2120 (2014).

44. M. G. Grabherr, B. J. Haas, M. Yassour, J. Z. Levin, D. A. Thompson, I. Amit, X. Adiconis, L. Fan, R. Raychowdhury, Q. Zeng, Z. Chen, E. Mauceli, N. Hacohen, A. Gnirke, N. Rhind, F. di Palma, B. W. Birren, C. Nusbaum, K. Lindblad-Toh, N. Friedman, A. Regev, Full-length transcriptome assembly from RNA-Seq data without a reference genome. *Nat. Biotechnol.* **29**, 644–652 (2011).

45. P. Gayral, J. Melo-Ferreira, S. Glémin, N. Bierne, M. Carneiro, B. Nabholz, J. M. Lourenco, P. C. Alves, M. Ballenghien, N. Faivre, K. Belkhir, V. Cahais, E. Loire, A. Bernard, N. Galtier, Reference-free population genomics from next-generation transcriptome data and the vertebrate-invertebrate gap. *PLOS Genet.* **9**, e1003457 (2013).

46. M. Patterson, T. Marschall, N. Pisanti, L. van Iersel, L. Stougie, G. W. Klau, A. Schönhuth, WhatsHap: Weighted haplotype assembly for future-generation sequencing reads. *J. Comput. Biol.* **22**, 498–509 (2015).

47. B. L. Browning, X. Tian, Y. Zhou, S. R. Browning, Fast two-stage phasing of large-scale sequence data. *Am. J. Hum. Genet.* **108**, 1880–1890 (2021).

48. J. Engelstädter, Asexual but not clonal: Evolutionary processes in automictic populations. *Genetics* **206**, 993–1009 (2017).

49. F. Prugnolle, T. De Meeus, Apparent high recombination rates in clonal parasitic organisms due to inappropriate sampling design. *Heredity* **104**, 135–140 (2010).

50. B. C. Haller, P. W. Messer, SLiM 3: Forward genetic simulations beyond the wright-fisher model. *Mol. Biol. Evol.* **36**, 632–637 (2019).

51. F. Rousset, *Genetic Structure and Selection in Subdivided Populations* (Princeton Univ. Press, 2004), vol. 40.

52. Wolfram-Research Inc., *Mathematica Edition: Version 8.0* (Wolfram Research, 2010).
